# A Comprehensive Survey of Enabling and Emerging Technologies for Social Distancing—Part II: Emerging Technologies and Open Issues

**DOI:** 10.1109/ACCESS.2020.3018124

**Published:** 2020-08-20

**Authors:** Cong T. Nguyen, Yuris Mulya Saputra, Nguyen Van Huynh, Ngoc-Tan Nguyen, Tran Viet Khoa, Bui Minh Tuan, Diep N. Nguyen, Dinh Thai Hoang, Thang X. Vu, Eryk Dutkiewicz, Symeon Chatzinotas, Björn Ottersten

**Affiliations:** 1 Department of Computer Science and EngineeringHo Chi Minh City University of Technology 117295 Ho Chi Minh City 700000 Vietnam; 2 Department of Computer Science and EngineeringVietnam National University-Ho Chi Minh City 54800 Ho Chi Minh City 700000 Vietnam; 3 School of Electrical and Data EngineeringUniversity of Technology Sydney 1994 Sydney NSW 2007 Australia; 4 Department of Electrical Engineering and InformaticsVocational CollegeUniversitas Gadjah Mada 59166 Yogyakarta 55281 Indonesia; 5 VNU University of Engineering and Technology, Vietnam National University Hanoi 711000 Vietnam; 6 Interdisciplinary Centre for Security, Reliability and TrustUniversity of Luxembourg 81872 4365 Luxembourg City Luxembourg

**Keywords:** Social distancing, pandemic, COVID-19, wireless, networking, positioning systems, AI, machine learning, data analytics, localization, privacy-preserving, scheduling, incentive mechanism

## Abstract

This two-part paper aims to provide a comprehensive survey on how emerging technologies, e.g., wireless and networking, artificial intelligence (AI) can enable, encourage, and even enforce social distancing practice. In Part I, an extensive background of social distancing is provided, and enabling wireless technologies are thoroughly surveyed. In this Part II, emerging technologies such as machine learning, computer vision, thermal, ultrasound, etc., are introduced. These technologies open many new solutions and directions to deal with problems in social distancing, e.g., symptom prediction, detection and monitoring quarantined people, and contact tracing. Finally, we discuss open issues and challenges (e.g., privacy-preserving, scheduling, and incentive mechanisms) in implementing social distancing in practice. As an example, instead of reacting with ad-hoc responses to COVID-19-like pandemics in the future, smart infrastructures (e.g., next-generation wireless systems like 6G, smart home/building, smart city, intelligent transportation systems) should incorporate a *pandemic mode* in their standard architectures/designs.

## I. Introduction

In the presence of contagious diseases such as the current COVID-19 pandemic, social distancing is an effective non-pharmaceutical approach to limit the disease transmission. By reducing the frequency and closeness of human physical contacts, social distancing can lower the probability of the disease transmission from an infected person to a healthy one, thereby significantly limiting the disease’s spread and severity. During the ongoing COVID-19 pandemic, many governments have implemented various social distancing measures such as travel restrictions, border control, public places closures, and quarantines. Nevertheless, the implementation of such aggressive and large-scale measures is facing significant challenges such as negative economic impacts, personal rights violation, difficulties in changing people’s behavior, and the difficulties arisen when there are many people staying at home. In such context, emerging technologies such as Artificial Intelligence (AI) can play a key role in addressing those challenges.

**FIGURE 1.**
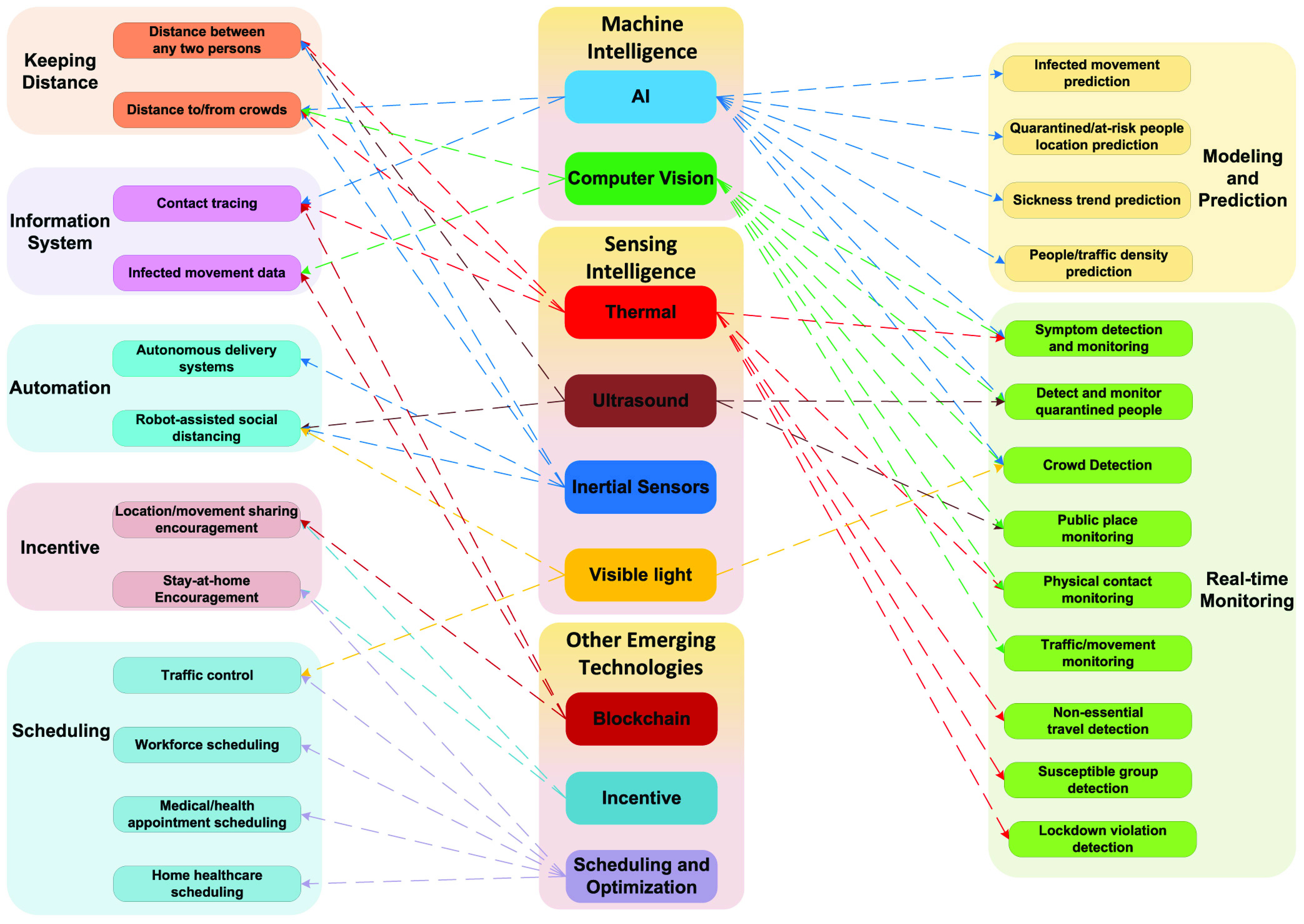
Application of technologies to different social distancing scenarios. Some technologies, e.g., AI and Thermal, can be applied to many scenarios, whereas technologies such as Visible Light and Ultrasound are applicable to fewer scenarios. Scenarios from the same group have the same color. The arrows that show the links from one technology to different scenarios have the same color.

In this two-part paper, we present a comprehensive survey of enabling and emerging technologies for social distancing. In Part I [1], we provide a comprehensive background on social distancing and how wireless technologies can be leveraged to enable, encourage, and enforce proper social distancing implementation. In this Part II, we discuss various emerging technologies, e.g., AI, thermal, computer vision, ultrasound, and visible light, which have been introduced recently in order to address many new issues related to social distancing, e.g., contact tracing, quarantined people detection and monitoring, and symptom prediction. For each technology, we have provided an overview, examined the state-of-the-art, and discussed how it can be utilized in different social distancing scenarios as illustrated in Fig. 1. Finally, some important open issues and challenges (e.g., privacy-preserving, scheduling, and incentive mechanisms) of implementing technologies for social distancing will be discussed. Furthermore, potential solutions together with future research directions are also highlighted and addressed.

**FIGURE 2.**
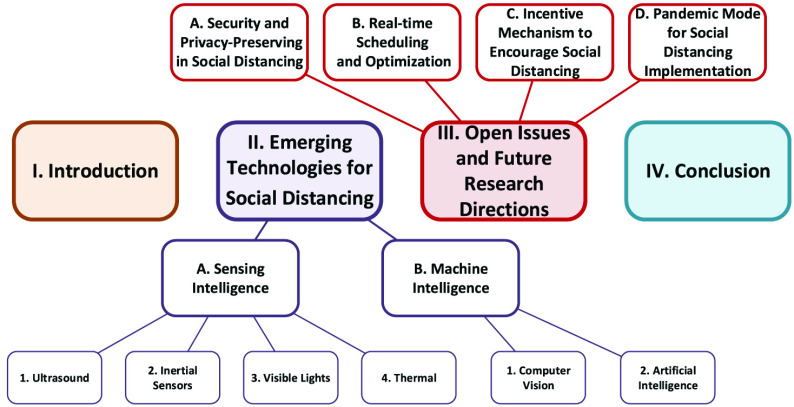
The organization of this (Part II) paper.

As illustrated in Fig. 2, the rest of this paper is organized as follows. We first discuss emerging technologies for social distancing in Section II. After that, we discuss open issues and future research directions of technology-enabled social distancing in Section III, and conclusions are given in Section IV.

## II. Emerging Technologies for Social Distancing

In addition to the wireless technologies, emerging technologies such as AI, computer vision, ultrasound, inertial sensors, visible lights, and thermal also can all contribute to facilitating social distancing. In this section, we categorize those technologies into sensing intelligence and machine intelligence technologies, provide a brief overview of each technology, and discuss how they can be applied for different social distancing scenarios.

### A. Sensing Intelligence

#### 1) Ultrasound

The ultrasound or ultrasonic positioning system (UPS) is usually used in the indoor environment with the accuracy of centimeters [6]. The system includes ultrasonic beacons (UBs) as tags or nodes attached to users and transceivers. Beacon units broadcast periodically ultrasonic pulses and radio frequency (RF) messages simultaneously with their unique ID numbers. Based on these pulses and messages, the receiver’s position can be determined by position calculation methods such as *trilateration* or *triangulation* [7]. In comparison with other RF-based ranging methods, the UPS does not require a line of sight between the transmitter and the receiver, and it also does not interfere with electromagnetic waves. However, since the propagation of the ultrasound wave is limited, most UPS applications for social distancing are only limited within the indoor environment.

##### a: Keeping Distance

For this purpose, UPS can be used to position and notify people. One of the first well known UPS systems is Active Bat (AB) [8] based on the time-of-flight of the ultrasonic pulse. Typically, an AB system consists of an ultrasonic receiver matrix located on the ceiling or wall, a transmitter attached to each target, and a centralized computation system to calculate the objects’ positions. As presented in [8], by using a receiver matrix with 16 sensors, the AB system can achieve very high positioning accuracy, i.e., less than 14 centimeters. However, a limitation of this system is its high complexity, especially if a large number of ultrasonic sensors are deployed.

**FIGURE 3.**
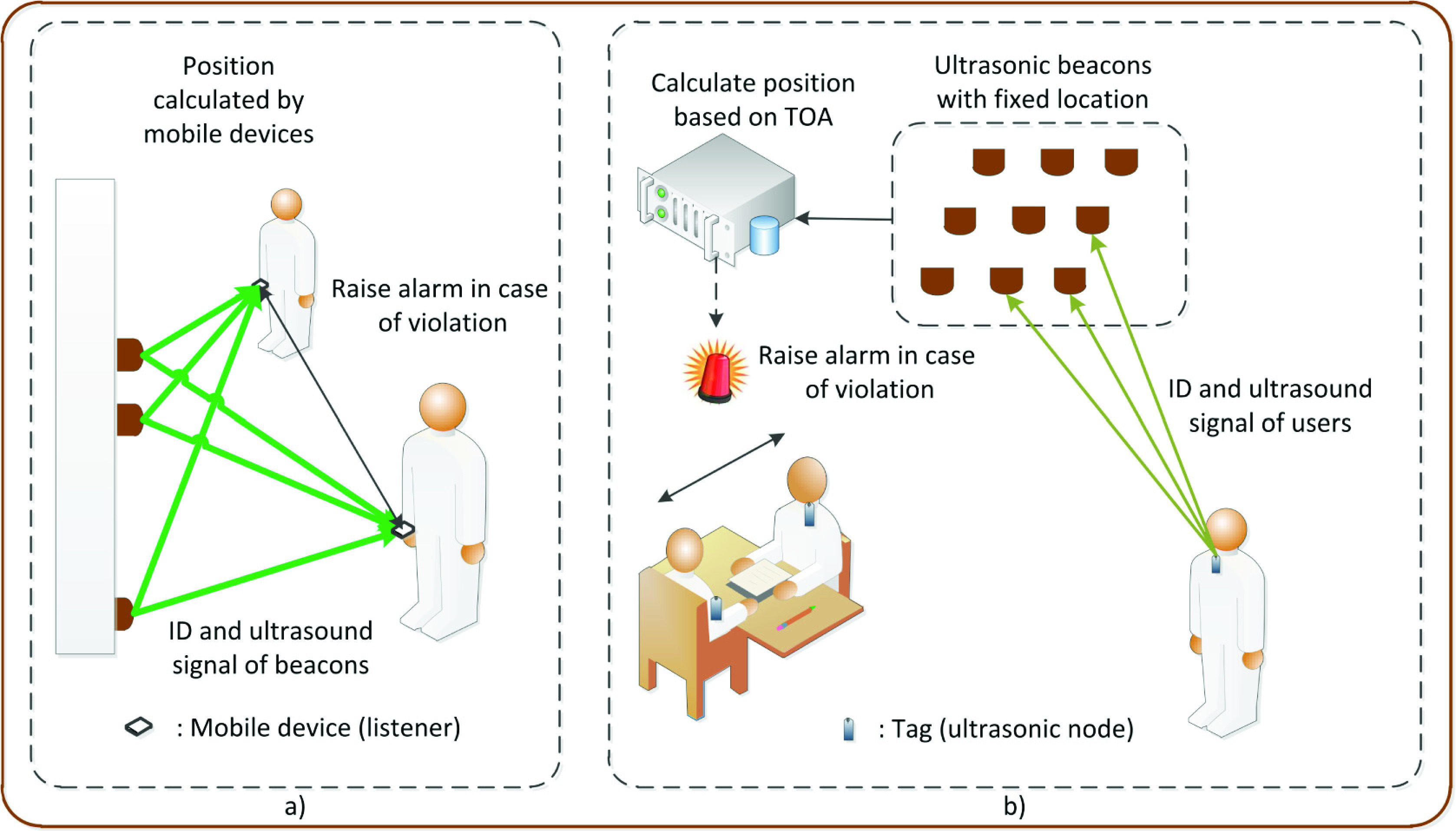
Ultrasound application for keeping distance using a) Cricket system [9], and b) Active Bat system [8]. The main difference between the two systems is that the user’s position is calculated by the user device in Cricket and by a central server in Active Bat.

Another limitation of the AB system is the privacy risk for users since the location of users under the AB system is calculated at the central server. To address that, the Cricket (CK) system is proposed in [9] wherein the position calculation is executed at the receivers. Specifically, a receiver in the CK system passively receives RF and ultrasound signals from UBs located on the wall or ceiling, and then the receiver calculates its position by itself based on UBs’ ID and coordinates. Since the receivers do not transmit any signals, the privacy of users will not be compromised. Fig. 3 demonstrates the two systems in the keeping distance application.

##### b: Real-Time Monitoring

In the context of social distancing, UPS can be an effective solution for real-time monitoring scenarios, especially gauging the number of people in public buildings. In particular, the main characteristic that makes UPS different from other positioning technologies is *confinement*, i.e., the ultrasound signal is confined within the same room as the UBs [7]. Among the other positioning technologies, only infrared technology shares the same characteristic. Nevertheless, infrared signals are prone to interference from sunlight and other thermal sources, and they also suffer from line-of-sight loss [7]. As a result, ultrasound is the most efficient technology for *binary positioning* [7], i.e., determine if the object is in the same room as the UBs or not. Thus, UPS can be particularly useful in the social distancing scenarios where the exact positions of people are not as necessary as the number of people inside a room (e.g., to limit the number of people). This technology is more efficient because it needs a few reference nodes (e.g., UBs) to determine the binary positions of people, which can significantly reduce implementation costs.

##### c: Automation

Ultrasound can also be applied in the social distancing scenarios that utilize medical robots or UAV. Mobile robots, especially medical robots, can play a key role in reducing the physical contact rates between the healthcare staff (e.g., doctors and nurses) and the patients inside a hospital, thereby maintaining a suitable social distancing level. In such scenarios, UPS can help to improve the navigation of medical robots. In [11], a navigation system based on Wi-Fi and ultrasound is proposed for indoor robot navigation. To deal with the uncertainties which are very common in crowded places like hospitals, the system employs a Partially Observable Markov Decision Process, and a novel algorithm is also introduced to minimize the calibration efforts.

In the social distancing context, besides outdoor applications, UAVs can also be employed to reduce the necessity of human physical presence. For example, UAVs can be used to deliver goods inside a building or to manage warehouse inventory. However, most of the existing studies focus on UAV navigation for the outdoor environment, which often relies on GNSS for UAV positioning. Since GNSS’s accuracy is low for the indoor environment, these methods cannot be applied directly for UAV navigation inside a building. To address that limitation, a navigation system is proposed in [10], which utilizes ultrasound, inertial sensors, GNSS, and cameras to provide precise (less than 10 cm) indoor navigation for multiple UAVs.

*Summary:* Ultrasound can be applied in several social distancing scenarios. In the keeping distance scenarios, UPS systems such as AB and CK can be applied directly to localize and notify people to keep a *safe* distance. Moreover, due to its confinement characteristic, ultrasound is one of the most efficient technology for binary positioning, which is particularly useful for monitoring and gauging the number of people inside the same room. In the automation scenarios, ultrasound can facilitate UAVs and medical robots navigations, especially for the indoor environment.

#### 2) Inertial Sensors

**FIGURE 4.**
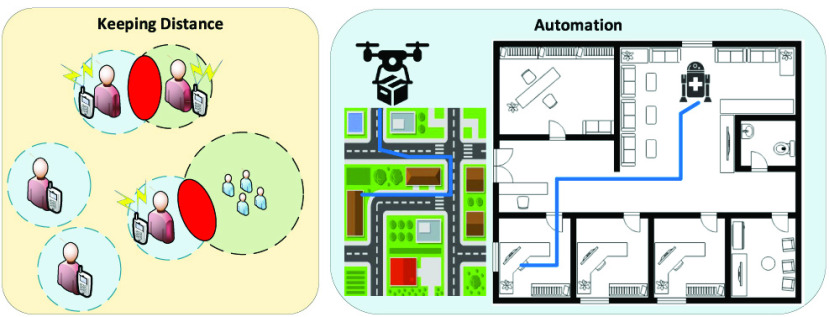
Inertial-sensors-based systems for several social distancing scenarios. In the keeping distance scenario, the built-in inertial sensors of smartphones can be utilized for user positioning. Based on this, the smartphone can warn the user when there are other users or crowds in close proximity. Inertial sensors can also help to localize and navigate UAVs and medical robots.

In the context of social distancing, inertial-sensors-based systems can be applied in distance keeping and automation scenarios as illustrated in Fig. 4. For example, positioning applications utilizing built-in inertial sensors can be developed for smartphones which can alert the users when they get close to each other or a crowd. Moreover, inertial sensors can be integrated into robots and vehicle positioning systems, which can facilitate autonomous delivery services and medical robot navigation. All of these scenarios can contribute to reducing the physical contact rate between people.

Inertial sensors consist of two special types of sensors, namely gyroscopes and accelerometers, attached to an object to measure its rotation and acceleration. Based on the measured rotation and acceleration data, the orientation and position displacements of the object can be determined [95]. Because inertial sensors do not require any external reference system to function, they have been one of the most common sensors for dead reckoning navigation systems, i.e., calculation of the current position is based on a previously determined position. Such navigation systems can provide accurate positioning within a short time frame. However, since the current position is determined based on the previously calculated positions, the errors accumulate over time, i.e., integration drift. Therefore, Inertial-Navigation-System (INS) is often used in combination with other positioning systems, e.g., GPS, to periodically reset the base position [95].

##### a: Keeping Distance

Traditionally, INS has been widely used for aviation, marine, and land vehicle navigation. Recently, the ever-increasing presence of smartphones has enabled many INS applications for pedestrian positioning and navigation, which can support social distancing scenarios. Moreover, INS is one of the few technologies that can enable accurate pedestrian positioning for the outdoor environment, especially when combined with other outdoor positioning technologies such as GPS. In [96], a smartphone-based positioning system is proposed. The system makes use of a smartphone’s built-in sensors, including gyroscopes, accelerometers, and magnetometers (sensors that measure magnetism), to calculate the smartphone’s position. In particular, magnetometers are combined with gyroscopes to improve accuracy of rotation measurements. This is done by correlating their measurements via a novel algorithm which uses four different thresholds to determine the weights of the gyroscope and magnetometers measurements in the correlation function. In [97], a wearable body sensors system using inertial sensors is proposed to measure the lower limb motion. The proposed system consists of three sensors attached to different parts of the human lower limb to measure its orientation, velocity, and position. Using this information and the initial position of a person, the person’s location can be tracked.

In [46], a novel indoor positioning system is developed using Wi-Fi and INS technologies. In this system, INS is utilized for the area where Wi-Fi coverage is limited, while Wi-Fi positioning is used to compensate INS’s integration drift. Another positioning system using inertial sensors and Wi-Fi is presented in [98], where Wi-Fi fingerprinting technique is used to improve the accuracy of the dead reckoning navigation. Because of the integration drift, a dead reckoning navigation system needs to frequently update its position by referencing to an external node. In the proposed system, a Wi-Fi fingerprinting map is set up in advance and the dead reckoning system can use the map to update its position. Moreover, in [46], the authors propose using Kalman filter to combine the measurement data from Wi-Fi and INS, which can reduce the error to 1.53 meters.

Besides Wi-Fi, INS can be used in combination with other positioning technologies. In [99] and [100], INS has been combined with the UWB technology for pedestrian positioning and tracking. Generally, INS helps to reduce UWB’s high implementation cost and complexity, while INS’s integration drift can be compensated. Particularly, INS is employed to compensate for the UWB’s low dynamic range and proneness to external radio disturbances in [99]. To enable the combination, an information fusion technique using the extended Kalman filter is proposed to fuse the measurement data coming from both the INS and UWB sensors. The result shows that the hybrid system can achieve better performance than both the individual systems. In [100], the information fusion problem between the INS and UWB is optimized to minimize the uncertainties in the measurements. As a result, the positioning accuracy can be significantly improved.

##### b: Automation

Besides pedestrian positioning, INS can also be applied for social distancing scenarios involving autonomous vehicles, e.g., medical robots and drone delivery. Generally, INS has been commonly used for medical robot applications, including surgeon assists, patient motion assists, and delivery robots. In this section, we will only focus on the medical and delivery robot applications for social distancing purposes. In [101], a novel INS system is developed specifically for mobile robot navigation. In addition, an error model is proposed to increase the accuracy of the involved inertial measurements. A Kalman filter is also proposed to precisely estimate the velocity and orientation of the robot in the presence of noises. A novel data fusion algorithm, leveraging an adaptive Kalman filter is presented in [102] for indoor robot positioning based on an INS/UWB hybrid system.

Unlike INS for mobile robots that are mostly developed for the indoor environment, INS for UAV focuses on outdoor applications. Note that UAV navigation must also consider its altitude, which adds more complexity. The authors of [103] leverage inertial sensors and cameras to determine the UAV’s position, velocity, and altitude. Particularly, the cameras attached to the UAV capture the images of the surrounding environment and send them to a control station. This station will then process the images to determine the UAV’s pose in regards to the surroundings. The pose’s data is then combined with the inertial sensors data via a Kalman filter to determine the UAV’s position and velocity. Similarly, a system combining inertial and vision sensors is developed in [104] for UAV positioning and navigation. The system utilizes two observers which have inertial and vision sensors. The first observer calculates the orientation based on gyroscope and vision sensors, and the second observer determines the position and velocity based on data from the accelerometers and vision sensors. The experimental results show that the vision sensors measurements can be used to compensate for the inertial sensors errors, thereby achieving a high accuracy even with low-cost inertial sensors.

*Summary:* The omnipresence of smartphones with built-in inertial sensors has opened many opportunities for developing positioning systems based on INS. For the distance keeping scenarios, INS positioning systems, especially for pedestrians, can play a vital role as they are readily available. In the other scenarios such as medical robot navigation and UAV delivery, INS-based techniques can help to increase the efficiency (more accurate path, and lower traveling time) of the existing navigation systems.

#### 3) Visible Light

The recent development in the light-emitting diodes (LEDs) technology has enabled the use of existing light infrastructures for communication and localization purposes due to attractive features of visible lights such as reliability, robustness, and security [12]–[14]. Visible light communication (VLC) systems usually comprise two major components, i.e., LED lights corresponding to transmitters to send necessary information (e.g., user data and positioning information) via visible lights and photodetectors (e.g., photodiodes) and imaging sensors (e.g., camera) playing the role of receivers [2]. Due to the ubiquitous presence of LED lights, VLC can be leveraged in many social distancing scenarios as discussed below.

##### a: Real-Time Monitoring

**FIGURE 5.**
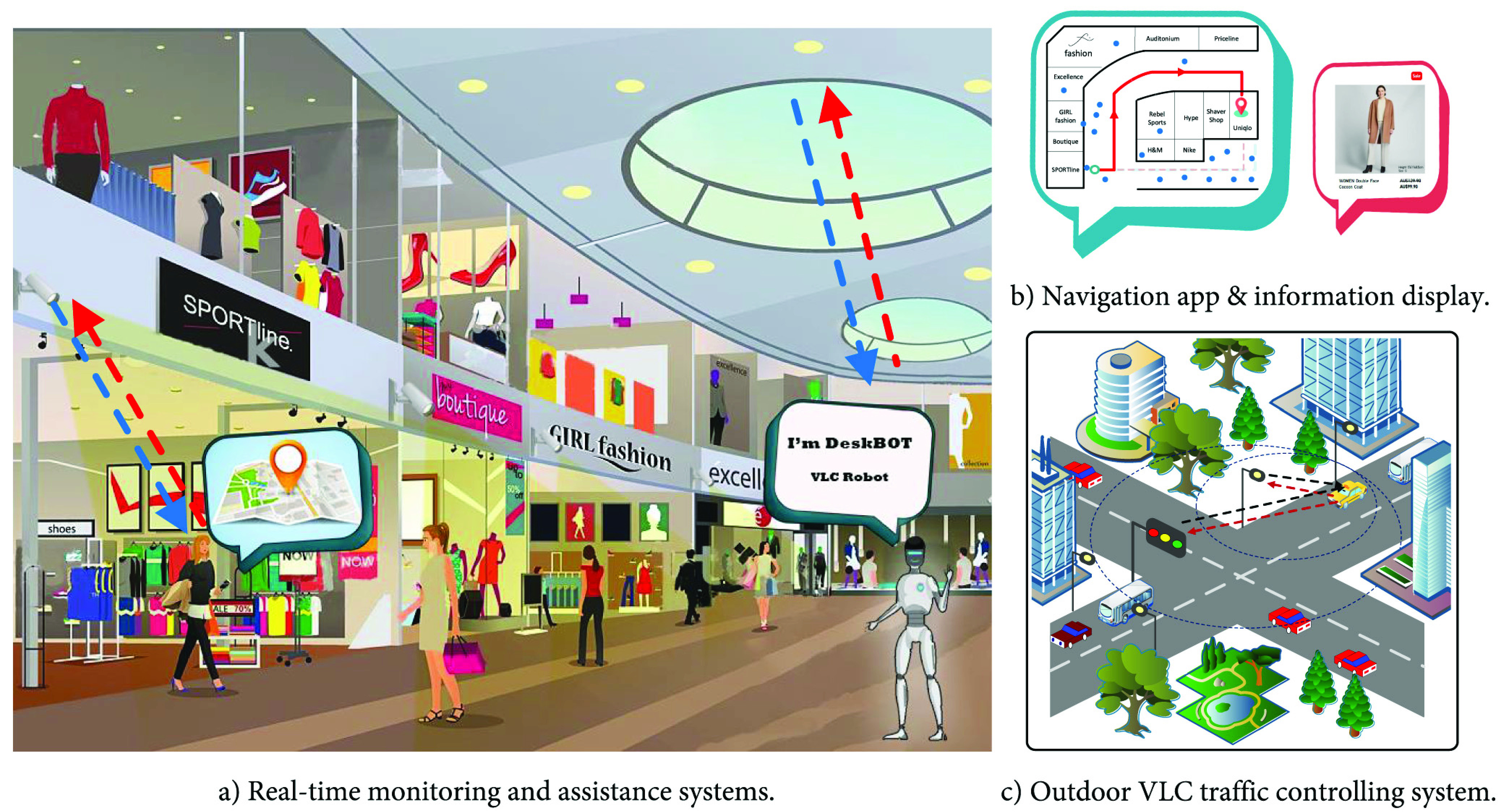
Visible light communications supporting social distancing in several scenarios. In indoor environments, visible light sensors can be utilized for real-time monitoring, information assistance system, and navigation application. For outdoor environments, visible light sensors can support traffic control.

Communication systems using visible light (e.g., LED-based communications) can provide precise navigation and localization solutions in indoor environments. Utilizing this technology, some applications can be implemented to support social distancing such as tracking individuals who are being quarantined, detecting and monitoring crowds in public places as shown in Fig. 5(a).

Due to many advantages such as low cost and ease of implementation, the VLC receiver using photodiodes can be employed as a “tag” that is integrated into mobile targets such as trolleys/shopping carts, autonomous robots, etc. People attached with these tags can perform self-positioning based on the *triangulation* method so that they can avoid crowded areas. Furthermore, the tags’ locations can be collected by the authorities to monitor people in public areas. Based on this location data, further actions can be carried out such as warning people by varying the color temperature of the lights in the crowded areas. It is worth noting that this solution will not reveal any personal information of users (e.g., customers) because it only requires communications between VLC-based tags and light fixtures. However, most VLC systems only provide half-duplex communications due to the fact that LED lights operate in the role of transmitters. Therefore, they should be combined with other wireless technologies like Bluetooth [15], [16], and Infrared [17] to enable an uplink communication with the server for location information exchange. Moreover, to improve the accuracy of positioning people in indoor environments using photodiodes, some advanced techniques can be used such as data fusion of AOA and RSS methods proposed in [18] and the AOA method using a multi-LED element lighting fixture introduced in [19]. One main disadvantage of the photodiode-based VLC systems is the need for hardware (i.e., the photodiode receiver) mounted on smart trolleys/shopping carts to receive light signals. Consequently, the system might fail to detect the locations of people who do not carry them. Nevertheless, pureLiFi company has recently invented a tiny optical front end which can be integrated into smartphones to take benefits of the photodiode receiver in high accuracy VLC-based localization services [20].

The rapid development of smartphones has enabled VLC-based applications on handheld devices such as indoor localization and navigation applications (e.g., smart retail systems [15], [16], [21]). These systems use front-facing cameras of mobile phones to receive visible light signals contained positioning information (e.g., the LED light’s ID or location) from visible light beacons [22]. The captured photos collected regularly by the front-facing camera are sent to a cloud/fog server for image processing to alleviate the computation on the phone. Then, the beacon’s ID and coordinates can be extracted and sent back to the phone. After that, the AoA algorithm is implemented to estimate the location and orientation of the phone. An attractive use case of the camera-based VLC systems [15], [16], [21] is to assist users to quickly find specific products in shopping malls or supermarkets. Thus, we can adopt this function to implement tracking and monitoring crowds in public places as well as assisting people in avoiding crowds in a proactive manner. It is worth noting that this solution is more convenient than using photodiodes since it uses front-facing cameras of smartphones as the VLC receivers, thus everyone using smartphones can be tracked. However, due to continuous photo shooting, these positioning applications are very energy-consuming, which is a major drawback of camera-based VLC systems when they are used for tracking people.

##### b: Automation

In public places, there is always a need for assistance in specific circumstances (e.g., information or physical supports for customers, older and disabled people). For instance, supporting staff in supermarkets can assist customers in finding products or help elder/disabled people to carry their goods. Similar assistance scenarios can be seen in hospitals, banks, and libraries. This results in an increase in close physical contacts between customers and assistants. Therefore, autonomous assistance systems using VLC technology can be employed to minimize the physical contacts as shown in Fig. 5(a) and (b).

Besides the navigation purpose, the smart retail systems [15], [16] can also provide information assistance services for shoppers. For example, the product description, sale information, or other necessary information can be displayed on the screen when the phone is under a certain LED light. Another example is information assistance services in museums [23], [24]. This can help to reduce the number of close physical contacts in these places.

Similar to the information assistance systems for reducing close physical contacts, autonomous robots using the VLC technology for communication and localization can also be deployed to assist people in certain circumstances, for example, elderly-assistant robots, walking-assistant robots, shopping-assistant robots, etc., [25], [26]. Moreover, visible light signals do not cause any interference to RF signals, and thus they can be effectively deployed in diverse indoor environments such as hospitals, schools, and workplaces.

##### c: Traffic Control

In the context of social distancing, high demand traffic can cause a large concentration of people in a certain area (e.g., city center). By adopting smart traffic light systems in [27], [28], we can deploy an intelligent traffic controlling system using the VLC technology to control large traffic flows as illustrated in Fig. 5(c). That can help to reduce vehicle density in public areas. The VLC technology provides a communication method between vehicles and the light infrastructure (e.g., traffic lights, street lights). First, vehicles can send their information (e.g., their IDs) to the light infrastructure by using its headlights as transmitters, thus the system can detect and monitor the traffic flow. However, in this case, it is required that the light infrastructure must be equipped with VLC receivers (e.g., traffic cameras or photodiodes). Second, based on the awareness of the traffic, the system can control the vehicles by sending instructions to guide them. In this case, the system uses traffic lights, or street lights as transmitters to send information and the vehicles use dash cameras to receive the information. For example, the system will notify them about hot zones that have a high density of vehicles and do not allow them to enter, so that they can avoid these zones.

*Summary:* The availability of smart retail systems is proof of the superior performance and convenience of VLC technology compared to other RF technologies in high precision indoor localization and navigation. By leveraging such commercial approaches, we can deploy the cost-effective crowd monitoring system on a large scale, not only in shopping malls or hypermarkets but also in other public places, such as airports, train stations, and hospitals, based on the existing illuminating infrastructures. Building/facilities managers can immediately alert or notify the users if they are in the middle of a crowd (e.g., varying the color temperature of the lights in the high-density zones). People can also take the initiative in planning their move to the desired locations without encountering the crowds. On the other hand, assistance systems help to reduce the number of staff/volunteers, nurses inside public buildings; or limit the close contacts between them and customers, patients. Moreover, the combination with other RF technologies such as Bluetooth and Infrared also ensures the location-based services are not interrupted when the smartphone is not being actively used by the user (e.g., the phone is in the pocket). Last but not least, the VLC technology can be a potential communication method between the intelligent traffic controlling system and vehicles in the outdoor environment. However, the main disadvantage of the VLC technology is that interference from ambient and sun lights have significant impacts on the visible light communication channels [12], [14]. It results in poor performance of the RSS-based positioning approaches and outdoor communications.

#### 4) Thermal

Thermal based positioning systems can be classified into two main categories which are infrared positioning (IRP) systems and thermal imaging camera (THC). Typical IRP systems such as [29], [31], [32] are low-cost, short-range (up to 10 meters) systems that use infrared (IR) signals to determine the position of targets via AOA or TOA measurement method. On the other hand, the THC, which constructs images from the object’s heat emission, can operate at a larger range (up to a few kilometers) [35]. Because of this difference, IRP and THC can be applied in different social distancing scenarios as discussed below.

##### a: Keeping Distance

In keeping distance scenarios, IRP systems such as Active Badge [31], Firefly [33], and OPTOTRAK [32] can be utilized. In the Active Badge, badges that periodically emit unique IR signals are attached to the targets. Based on the distances from the fixed infrared sensors to the badges, the target’s position can be calculated. As a result, this application can be useful to determine the distance between two people as well as to identify crowds in indoor environments. The main advantages of this solution are low cost and easy implementation. However, it requires users to wear tag devices to track their locations.

To achieve a higher positioning accuracy, the Firefly [33] and OPTOTRAK [32] systems can be implemented. These systems contain infrared camera arrays and infrared transmitter called markers. Due to the difference in setups (one target is attached with one tag in Firefly and multiple tags in OPTOTRAK), the Firefly system can accurately determine the target’s 3D position, whereas the OPTOTRAK system can capture the target’s movement. The main disadvantage of these systems is that they are prone to interference from other radiation sources such as sunlight and light bulbs. Combined with their short-range, IRP is mostly applicable in small rooms with poor-light conditions.

##### b: Physical Contact Monitoring

**FIGURE 6.**
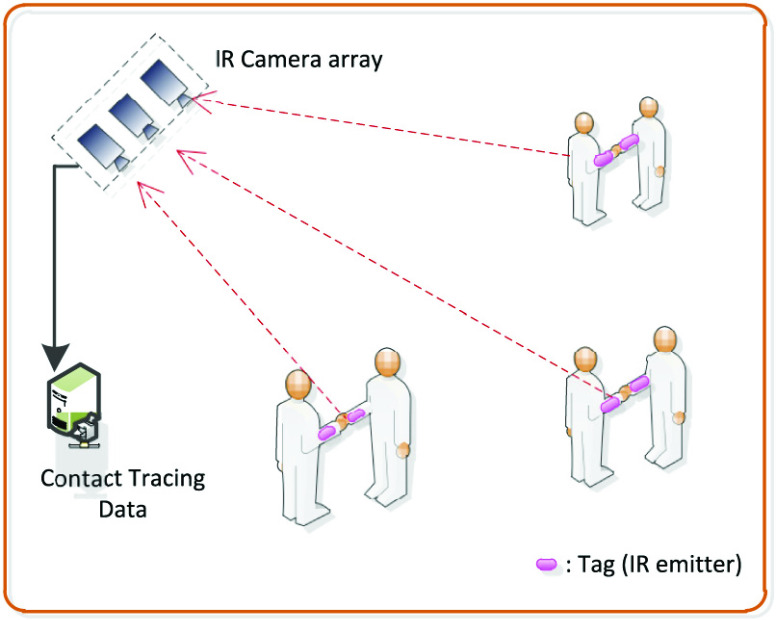
Physical contact monitoring by infrared system [33]. IR cameras can be utilized to detect and monitor physical contact among people. If there is close contact between any two people, the event can be recorded for future usage, e.g., contact tracing.

Since the Firefly and OPTOTRAK systems can accurately capture movements, they can be useful for contact tracing scenarios in social distancing. For example, markers can be attached to the target’s body parts which are usually used in physical contacts, e.g., hands for handshakes and body for hugs. The movement of these body parts can then be captured by the IR camera as illustrated in Fig. 6, and the recorded data can be analyzed later to determine if there are close contacts between the target and other people. Based on this information, the contacts that the target made can be traced later if necessary.

**FIGURE 7.**
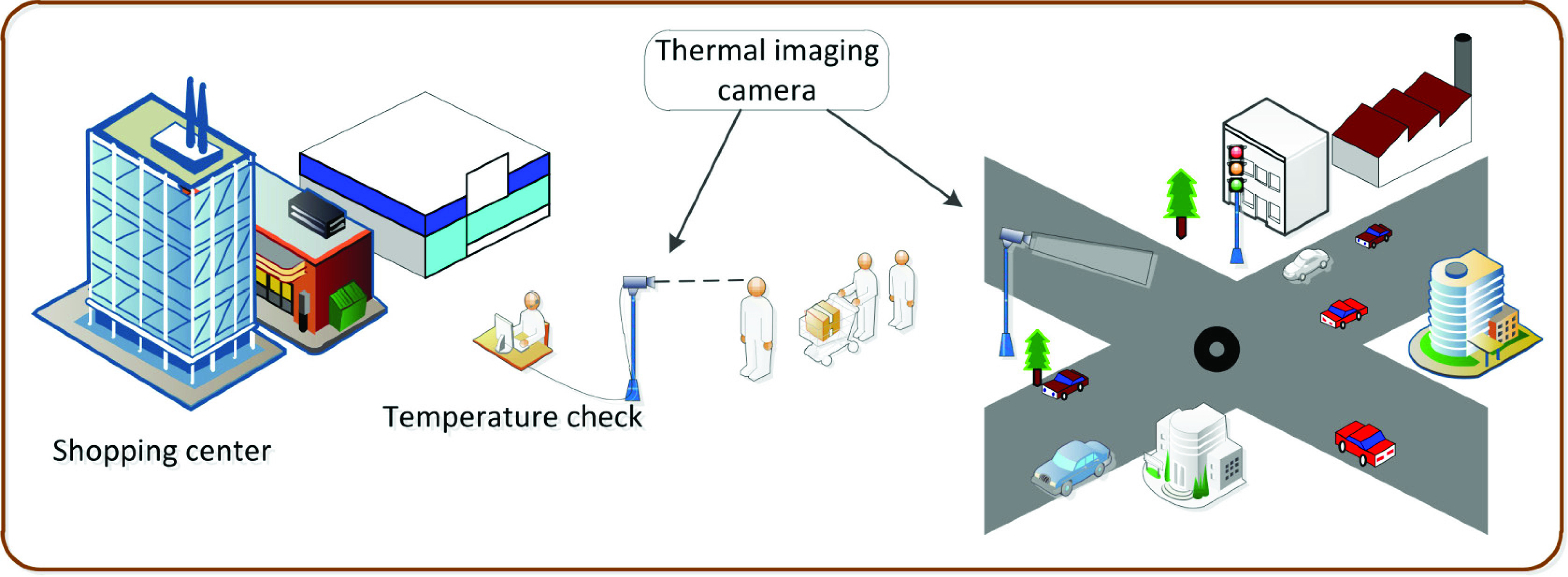
Thermal cameras used in susceptible group detection and traffic monitoring. Thermal cameras can be used to check body temperature, thereby detecting susceptible groups and people with symptoms. For traffic monitoring, both infrared positioning systems and thermal cameras can be utilized, especially in poor-light conditions, e.g., at night.

##### c: Real-Time Monitoring

For traffic monitoring in social distancing contexts, both IRP and THC can be utilized, especially in poor-light conditions. The authors in [34] propose a robust vehicle detector based on the IRP under the condition to quantify traffic level and flow. The collected data can be sent to assist the authorities in social distancing monitoring. However, since IRP has a short range, THC systems such as [37] can be a better choice in a larger area with high vehicle density.

Due to its very high observation range (a few kilometers) [36]–[38], THC is particularly effective for real-time monitoring scenarios, such as public building monitoring, detecting closure violation, and non-essential travel detection, which does not require high positioning accuracy. THC systems such as those proposed in [29], [30] are efficient in these scenarios since they are light-weight and can cover a wide area with medium accuracy.

Another application of thermal technology is to detect susceptible groups. Since the THCs measure heat emitted from people or other objects, they can be used for checking people’s temperature quickly from a far distance [39], [40]. Further, the THC system has the ability to detect slight temperature differences with a resolution of 0.01 degrees [41]. Thus, it can be a good means to check health conditions and sickness trends of patients. Moreover, the system can be deployed in shopping centers to measure customers’ temperature remotely. This can help to detect infection symptoms early and also prevent the disease spread.

*Summary:* Thermal based positioning systems are helpful in some social distancing scenarios, especially in poor-light conditions. For short-range communication applications, the IRP is cost-effective and can be used for positioning and tracing purposes. Whereas, some light-weight THC systems can be leveraged for real-time monitoring over long distances due to their high range. However, the high cost of THC should be considered when implementing THCs in practice.

Table 1 provides a summary of the surveyed sensing intelligence technologies. Generally, each technology has a special characteristic that makes it a very effective solution for a specific scenario. For example, ultrasound signals are confined by walls, which enables low-cost ultrasonic positioning system to efficiently monitor people in a small room. Furthermore, since inertial sensors are built-in in most smartphones, they can be quickly utilized for keeping distance in smartphone applications. In addition, visible light technology can be leveraged for building information assistance systems which help to reduce human presence. Finally, thermal camera is the only technology that can detect people over a large distance (a few kilometers) without the need for attached devices, which makes it an ideal solution to detect violation of quarantines or closures.

**TABLE 1:** Summary of Sensing Intelligence Technologies Applications to Social Distancing

Technology	Range	Accuracy	Cost	Privacy	Scalability	Indoor/Outdoor	Readily available devices
Ultrasound [7], [8], [10], [11]	Short, confined by walls	Less than 14 cm [8]	Low to Medium	Low [8] to High [9]	Medium	Indoor	Active Bat, Cricket systems
Inertial sensors [46], [95], [96], [98], [101], [105]	Not applicable	Less than 1 m [95]	Low	High	High	Both	Smartphone, body sensors networks
Visible light [15], [16], [18], [20], [21], [24]–[26]	Short	≤ 1 cm [25], ≤ 10 cm [16], [18], [26], ≤ 20 cm [19]	Low to Medium	High	High	Both	Smartphone, Smart Retail
Thermal [33], [35]–[37], [39]	IRP 10 m, THC a few km	IRP ≤ 0.125 mm [32], THC ≤ 0.9 m [38]	Medium to High	Low to High	Low to Medium	Both	Thermal Cameras

### B. Machine Intelligence

#### 1) Computer Vision

Computer vision technology trains computers to interpret and understand visual data such as digital images or videos. Thanks to recent breakthroughs in AI (e.g., in pattern recognition and deep learning), computer vision has enabled computers to accurately identify and classify objects [48]. Such capabilities can play an important role in enabling, encouraging, and enforcing social distancing. For example, computer vision can turn surveillance cameras into “smart” cameras which can not only monitor people but also can detect, recognize, and identify whether people comply with social distancing requirements or not. In this section, we discuss several social distancing scenarios where computer vision technology can be leveraged, including public place monitoring, and high-risk people (quarantined people and people with symptoms) monitoring and detection.

##### a: Public Place Monitoring

**FIGURE 8.**
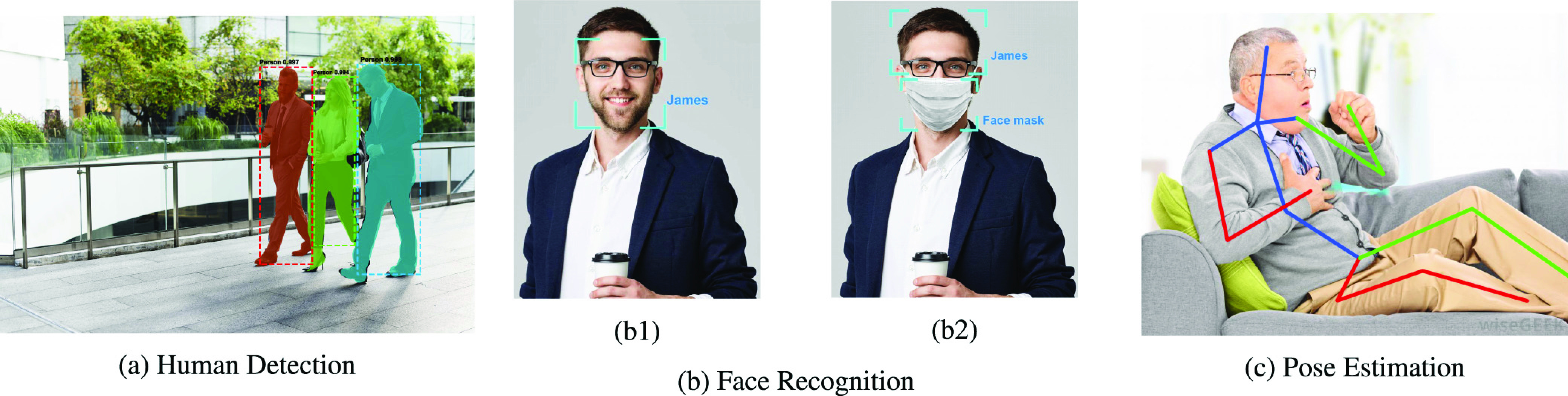
Computer vision technologies for social distancing: (a) human detection to identify the number of people in the public place [51], (b) face recognition to identify (b1) the full face of isolated person, (b2) person with mask or person behind the mask [52], and (c) pose estimation to detect one with coughing symptom [53].

Despite government restrictions and recommendations about social gathering, some people still do not comply with them, which can cause the virus infection to the community. In such context, human detection features in object detection [49], a major sub-field of computer vision, can help to detect crowds in public areas through real-time images from surveillance cameras. An example scenario is described in Fig. 8(a). If the number of people in an area does not meet the social distancing requirement (e.g., gathering above 10 people), the authorities can be notified to take appropriate actions.

There are two main approaches to detect humans from images in object detection namely region-based and unified-based techniques. The former detects humans from images in two stages including the region proposal and the processing according to the regions [50]. Based on this approach, several frameworks including Fast-RCNN [56] and Faster-RCNN [57] are developed in combination with Convolution Neural Network (CNN) [54]. In [58], the authors improve the Faster-RCNN by proposing the Mask Regions with the CNN features (Mask RCNN) method which masks the bounding box to detect the object with high accuracy while adding a minor overhead to the Faster-RCNN. Mask RCNN outperforms previous methods by simplifying the training process and improving the accuracy in detecting humans in the images for calculating the density of people in a particular area.

Although the above region-based approach has high recognition accuracy [58], it has high complexity, which is unsuitable for devices with limited computational capacity. To address this, the unified approach is more appropriate to implement, which can reduce the computational complexity by detecting humans from images with only one step. This approach maps the pixels from the image to the bounding box grid and class probabilities to detect humans or objects in real-time. Following this direction, the You Only Look Once (YOLO) method proposed in [59] can detect/predict objects (even small ones) in real-time with high accuracy. In addition, in [60], the authors propose the Single Shot Multibox Detector (SSD) framework which uses a convolution network on the image to calculate a feature map and then predict the bounding box. Through experimental results, they demonstrate that this method can detect objects faster and more accurately than those of both YOLO and Faster-RCNN. For public place monitoring, both YOLO [59] and SSD [60] can be used to detect fast and accurately humans from real-time images or videos of surveillance cameras. After identifying people, we can use a real-time automatic counter to count and identify whether the number of gathering people is complying with social distancing requirements or not.

##### b: Detecting and Monitoring Quarantined People

To prevent the spread of the virus from an infected person to others, the infected person or people who had physical contact with them must be isolated at the restricted areas or at home. For example, people who come back from highly infected countries/regions of COVID-19 are often requested to be quarantined or self-isolate for 14 days. Due to the lack of facilities, most countries require these people to self-isolate at home. In this case, the face recognition capability of computer vision can help to enforce this requirement by analyzing the images or videos from cameras to identify these people (i.e., to check whether they breach the self-isolation requirements or not). If these people are detected in public, the authorities can be notified to take appropriate actions.

Unlike object detection, the dataset including the full face images of the isolated people needs to be built. The face recognition system firstly learns from this dataset and then analyzes the images from public surveillance cameras to identify their appearances as in Fig. 8(b1). The authors in [61] propose a framework named DeepFace using Deep Neutral Network (DNN) which can detect with an accuracy of 97.35% and 91.4% in Labeled Faces in the Wild (LFW) and YouTube Faces (YTF) dataset, respectively. To improve the accuracy in detecting humans from surveillance cameras, some advanced techniques can be implemented such as [62], [63] and [64].

To prevent the spread of infectious diseases such as COVID-19, people are often required to wear masks in public places, which necessitates approaches to recognize or identify people with or without masks as illustrated in Fig. 8(b2). For example, the cameras in front of a public building can recognize and send warning messages (e.g., a beep sound) to remind the person who does not wear a mask when he/she intends to get into the building. This idea is introduced in [70] by using CNN to detect people who do not wear the masks. However, this work is just at the first step, which still requires much more efforts to demonstrate the effectiveness as well as improve the accuracy.

##### c: Symptoms Detection and Monitoring

After a few days of being infected with the virus, the infected person may have some symptoms such as coughing or sneezing. To minimize spreading the virus to others, it would be very helpful if we can detect these symptoms from people in public and inform them or the authorities. The idea here is similar to that of using thermal imaging cameras at airports or train stations. Specifically, detecting human behaviors, motion, and pose in computer vision can play a pivotal role [65]. Pose estimation captures a person with different parts (as illustrated in Fig. 8(c)) then detects human behaviors by studying the parts’ movements and their correlation. For example, a coughing person in Fig. 8(c) usually moves his hand near his head, and his head would have a vibration.

Recognition of human behaviors from surveillance cameras is a challenging problem because the same behaviors may have different implications, depending on the relationship with the context and other movements [66]. The recent advances in AI/ML are instrumental in correlating different movements/parts to interpret the associated behavior. In [67] the authors propose to use CNN [54] to enhance the accuracy of the model of the interaction between different body parts. In addition, the authors in [68] introduce several methods to detect body parts of multiple people in 2D images, and the authors in [69] propose methods to estimate 3D poses from matching of 2D pose estimation with a 3D pose library. These works can be further developed for future studies to detect people with symptoms of the disease such as coughing or sneezing in real-time. To improve the accuracy of the symptom detection in social distancing, computer vision-based behavior detection methods can be combined with other technologies, e.g., thermal imaging.

##### d: Infected Movement Data

To prevent the spread of the virus, tracing the path of an infected person plays an important role in finding out the people who were in the same place as the infected person. For this purpose, computer vision technology can not only detect infected people by facial recognition but also contribute to the positioning process. In [43], the movement of people is determined by analyzing the key point of transition frames captured from smartphone cameras. This method can draw the trajectory of movements and the location with an accuracy around two meters. In [44], the authors propose to combine the human detection techniques of computer vision with digital map information to improve the accuracy. In this study, the user path from cameras is mapped to the digital map which has the GPS coordinates. This method can achieve a very high accuracy within two meters. In another approach, the authors in [45] propose to use both smartphones’ cameras and inertial-sensor-based systems to accurately localize targets (with only 6.9 cm error). This approach uses the fusion of keypoints and squared planar markers to enhance the accuracy of cameras to compensate for the errors of inertial sensors.

##### e: Keeping Distance

Computer vision can also be very helpful to support people in keeping distance to/from the crowds. In [42], the authors develop an on-device machine-learning-based system leveraging radar sensors and cameras of a smartphone. When the radar sensor detects the surrounding moving objects, the smartphone camera can be utilized to capture its surrounding environment. Taking into account the recorded data, the smartphone can train the data using machine learning algorithms to determine the existence of nearby people and its distance from those people with respect to the social distancing requirements. We can also use a smartphone to estimate the distance between the mobile user and other people using radar sensors and cameras along with machine learning algorithms.

*Summary:* Computer vision can be utilized in several social distancing scenarios, especially the ones that require people monitoring and detection. Particularly, computer vision is the only method that can differentiate between people and identify complex features such as masks and symptoms. To further improve the effectiveness of computer vision in the social distancing context, future research should focus on increasing the accuracy and reducing the complexity of computer vision methods, so that they can be integrated into existing systems such as surveillance cameras.

#### 2) Artificial Intelligence

Over the last 10 years, we have witnessed numerous applications of AI in many aspects of our lives such as healthcare, automotive, economics, and computer networks [106]. The outstanding feature of AI technologies is the ability to automatically “learn” useful information from the obtained data. This leads to more intelligent automation, operating cost reduction as well as the great compatibility to adapt to changing environments. For that, AI (and its underlying machine learning algorithms) can also play a key role in social distancing, especially in modern lives, with many practical applications, as discussed below.

##### a: Distance To/From Crowds and Contact Tracing

**FIGURE 9.**
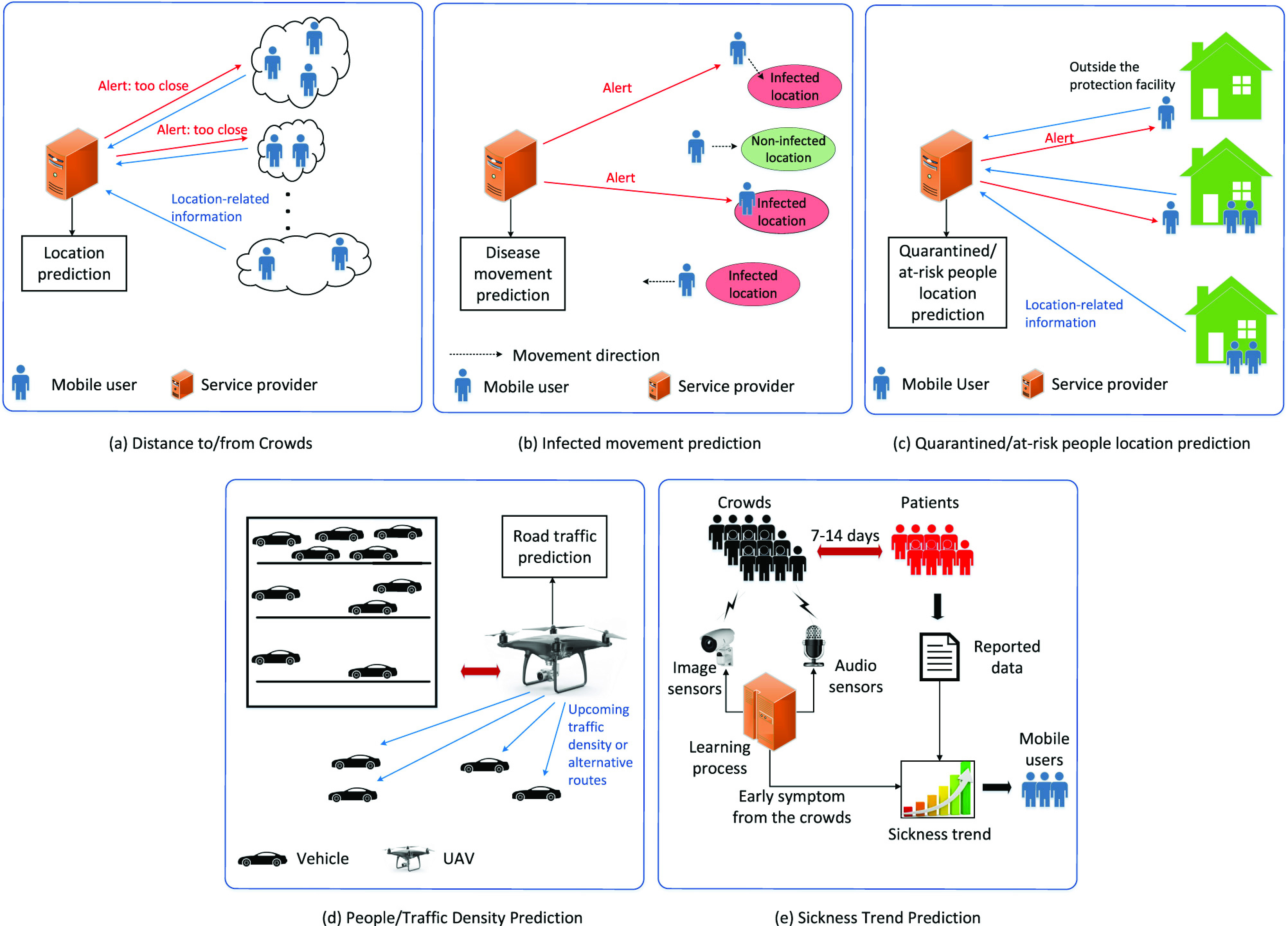
Application of artificial intelligence to several social distancing scenarios. By predicting people location, AI can be utilized to warn people to avoid potential crowded places in (a) distance/to from crowds scenario, alert people about infected locations in (b) infected movement prediction scenario, detect quarantine violation in (c) quarantined/At-Risk people location prediction. AI can also be leveraged to warn people or propose alternate routes in (d) people/traffic density prediction. Using data from audio and image sensors, AI can predict potential infected places before the real disease occurs in (e) sickness trend prediction.

Applications of machine learning to users’ location data allow us to effectively monitor the distance between people and trace the close contacts of infected people. In [150], the authors analyze the accuracy of a user’s location prediction based on his/her friends’ location datasets. In this case, a temporal-spatial Bayesian model is developed to select influential friends considering their influence levels to the user. Thus, the service provider can predict the exact location of a mobile user by using the temporal-spatial Bayesian model. Then, when the user is too close to other mobile users/people at crowded public places or his/her friends when they go in a group as illustrated in Fig. 9(a), his/her smartphone can alert to keep a safe distance. In addition, using the list of influential friends based on their ranks, the service provider can utilize it for the contact tracing purpose when the mobile user or one of his/her influential friends in the list gets infected. Moreover, the local-experts-finding scheme proposed in [151] can be utilized to find the local social media users of a certain area. Based on this, information such as current crowds locations can be extracted more efficiently.

##### b: Infected Movement Prediction

Another application of machine learning is to predict infected people movement from one location to another one and hence can potentially predict the geographic movement of the disease. The prediction is particularly crucial as infected people may travel to various places and can accidentally infect others before know that they carry the disease. In [152], the authors introduce a smartphone-based location recognition and prediction model to detect the current location and predict the destination of mobile users. In particular, the location recognition is implemented using the combination of k-nearest neighbor and decision tree learning algorithms, and the destination prediction is realized using hidden Markov models. Given the history of infected people movement, we can adopt the above model to recognize and predict the potential geographic movement of the disease. Using the information, people can be advised to stay away from the possible infected locations through alerts from their smartphones as illustrated in Fig. 9(b).

##### c: Quarantined/at-Risk People Location Prediction

The current location prediction of quarantined people, e.g., infected people, and at-risk people, e.g., sick and elder people, is very important to monitor whether they currently stay at the self-quarantined and self-protection areas, e.g., their homes, or not. To this end, a machine-learning-based location prediction approach can help to detect the current position of those people in a certain area. In [153], the authors apply the auto-encoder neural networks and one-class support vector machines to verify whether a user is within a specific area or not. Considering various channel models, i.e., path-loss, shadowing, and fading, the proposed solutions can achieve Neyman-Pearson optimal performance by observing the probability of miss-detections and false-alarms. The authors in [154] propose a novel localization system leveraging the federated learning to allow mobile users to collaboratively provide accurate location services without revealing mobile users’ private location. As such, the authors utilize deep neural networks with the Gaussian process to accurately predict the desired location of the mobile users. As a result, we can apply the proposed solutions to detect if infected people or at-risk people currently move away from their homes as illustrated in Fig. 9(c). Moreover, we can utilize the proposed solutions to determine the movement frequency of the self-isolated people outside the protection facility. Using the movement frequency history, the authorities can enforce them to stay at the protection facility for further infection prevention.

##### d: People/Traffic Density Prediction

Predicting the density of people or the number of people in public places allows us to efficiently schedule or guide people to stay away or refrain from coming to soon-to-be over-crowded places. For example, when the predicted number of people in a certain place almost reaches a predefined threshold (e.g., according to the social distancing requirement), the service provider can broadcast a local notification to incoming people via cellular networks, aiming at encouraging them to move to another area. In [155], the authors adopt advanced machine-learning-based approaches for edge networks to predict the number of mobile users within base stations’ coverages. Particularly, the framework first groups the base stations into clusters according to their network data and deployment locations. Then, using various machine learning algorithms, e.g., the Bayesian ridge regressor, the Gaussian process regressor, and the random forest regressor, we can predict the number of mobile users within their network coverages. From the preceding architecture, one can utilize Wi-Fi hotspots and cluster them based on their locations. By doing so, we can predict the number of people within each cluster’s coverage. Using the same architecture, we can extend the application to predict the traffic level on the roads. Specifically, upon predicting the number of vehicular users on the roads, we guide the drivers to choose particular routes to satisfy the social distancing requirements, e.g., suggest alternative routes to avoid crowded areas. In [156], the authors introduce a UAV-enabled intelligent transportation system to predict road traffic conditions using the combination of convolutional and recurrent neural networks. In particular, sensor cameras on the UAVs are utilized to capture the current road traffic. By using this information, the UAVs can then predict the road traffic conditions using the aforementioned deep learning methods. Thus, from the traffic prediction, the UAVs can work as mobile road-side units to orchestrate road traffic for over-crowding avoidance by informing the upcoming road traffic conditions to vehicular users via cellular networks accordingly (Fig. 9(d)).

##### e: Sickness Trend Prediction

Machine-learning-based location prediction method is also of importance to predict the sickness trend in specific areas. This sickness trend prediction can be used to inform people to stay safe from possible infected places. For example, the work in [157] designs a contactless surveillance framework, i.e., FluSense, to predict the influenza-like disease 7–14 days before the real disease occurs in the hospital waiting areas. In particular, a set of real-time sensors including a microphone array to detect normal speech/cough sounds and a thermal camera to detect crowd density are embedded into an edge computing platform. Considering millions of non-speech audio samples and hundred thousands of thermal images for audio and image recognition models, the proposed framework can accurately predict the number of daily influenza-like patients with Pearson correlation coefficient of 0.95. The prediction model from this work can be correlated/combined with the localized medical/health information (e.g., from local hospitals/clinics) to further improve the prediction accuracy as shown in Fig. 9(e). We can then inform the local mobile users about the sickness trend prediction to avoid the potential areas where many influenza-like patients exist.

##### f: Symptom Detection and Monitoring

Coughing is one of the most common and detectable symptoms of influenza pandemics. In the presence of a pandemic, the early detection of such symptoms can play a key role in limiting the disease spread from the infected to the susceptible population. For example, if a coughing person can be detected and identified in public places, that person and the people in close proximity can be tested for the disease.

In several studies, such as [158]–[161], AI technologies are leveraged to identify the cough patterns in audio recordings collected from microphones or acoustic sensors. In [158], audio signals are analyzed using recurrent and convolutional neural networks to detect coughs with high accuracy (up to 92%). Similarly, a hidden Markov model is proposed in [159] to detect cough from continuous audio recordings. In addition to audio signals, signals from motion sensors are also analyzed in [160] by a novel classification algorithm. However, a common limitation of these approaches is that they require the sensors to be attached to the person, which is not always possible in social distancing scenarios. To address this problem, a cough detection system is proposed in [161]. This system utilizes a wireless acoustic sensors network connected to a central server for both cough detection and localization. In particular, when a sound is detected, the sensors first localize the sound source by the AOA technique. Then, the sensors send the measured sound signals to the central server for cough identification using a novel classification algorithm. In the social distancing context, this system can be applied directly to monitor and detect coughing people in public places. Nevertheless, a limitation of this system is that the localization and measurement errors increase significantly when the sound source is too far from the sensors. Besides coughs, other physiological metrics such as cardiovascular activity, body temperature, and respiration can also be meaningful indicators. Especially, based on those metrics, an infected case can be potentially detected before clinical symptoms, e.g., fever, occur [162]. However, early detection algorithms need to be developed specifically for COVID-19.

*Summary:* Various AI technologies can be leveraged to facilitate social distancing implementations, especially in the scenarios that require modeling and prediction. In particular, AI technology can help to predict people’s locations, traffic density, and sickness trends. Moreover, AI-based classifications algorithms can be utilized to detect symptoms such as coughs in public areas.

## III. Open Issues and Future Research Directions

In this section, we discuss the open issues of social distancing implementation such as security and privacy concerns, social distancing encouragement, work-from-home, and the increased demands in healthcare appointments, home healthcare services, and online services. To addressed these issues, potential solutions are also presented.

### A. Security and Privacy-Preserving in Social Distancing

Most aforementioned social distancing scenarios (see Table 1 for more details) call for people’s private information, to a different extent, ranging from their face/appearance to location, travel records, or health condition/data. These data, if not protected properly, attract cyber attackers and can turn users into victims of financial, criminal frauds, and privacy violation [125]. Users’ data like health conditions can also adversely impact people’s employment opportunities or insurance policy. Given that, to enable technology-based social distancing, it is critical to develop privacy-preserving and cybersecurity solutions to ensure that users’ private data are properly used and protected.

The general principle of users’ privacy-preserving is to keep each individual user’s sensitive information private when the available data are being publicly accessed. To do so, data privacy-preserving mechanisms including data anonymization, randomization, and aggregation can be utilized [116]. For example, Apple, Google, and Facebook have developed people mobility trend reports while preserving users’ privacy during the COVID-19 outbreak. In particular, Apple utilizes random and rotating identifiers to preserve mobile users’ movements privacy [119]. Meanwhile, Google aggregates and uses anonymized datasets from mobile users who turn on their location history settings in their Android smartphones. In this case, a differential privacy approach is applied by adding random noise to the location dataset with the aim to mask individual identification of a mobile user [117]. Similarly, Facebook utilizes aggregated and anonymized user mobility datasets and maps to determine the mobility trend in certain areas including the social connectedness intensity among nearby locations [118]. In addition to the Apple’s, Google’s, and Facebook’s latest privacy-preserving implementation, in the following, we will thoroughly discuss how the latest advances in security and privacy-preserving techniques can help to facilitate social distancing without compromising users’ interest/privacy.

#### 1) Location Information Protection

To protect the exact location/trajectory information of participating mobile users in social distancing, some advanced location-based privacy protection methods can be adopted. Specifically, we can anonymize/randomize/obfuscate/perturb the exact location of each mobile user to avoid malicious attacks from the attackers using the following mechanisms. For example, the authors in [126] develop a privacy-preserving location-based framework to anonymize spatio-temporal trajectory datasets utilizing machine-learning-based anonymization (MLA). In this case, the framework applies the $K$-means machine learning algorithm to cluster the trajectories from real-world GPS datasets and ensure the $K$-anonymity for high-sensitive datasets. Using the $K$-anonymity [127], [128], the framework can collect location information from $K$ mobile users within a cloaking region, i.e., the region where the mobile users’ exact locations are hidden [129], [130]. In [131], the use of $K$-anonymity is extended into a continuous network location privacy anonymity, i.e., $KDT$-anonymity, which not only considers the average anonymity size $K$, but also takes the average distance deviation $D$ and the anonymity duration $T$ into account. Leveraging those three metrics, the mobile users under realistic vehicle mobility conditions can control the changes of anonymity and distance deviation magnitudes over time.

The authors of [132] propose a mutually obfuscating paths method which allows the vehicles to securely update accurate real-time location to a location-based service server in the vehicular network. In this case, the vehicles first hide their IP addresses due to the default network address translation operated by mobile Internet service providers. Then, they generate fake path segments that separate from the vehicles’ actual paths to prevent the location-based service server from tracking the vehicles. Exploiting dedicated short-range communications (DSRC) among vehicles and road navigation information from the GPS, the vehicles can mutually generate made-up location updates with each other when they communicate with the location-based service server (to obtain spatio-temporal-related information). In [133], vehicles which use location-based services can dynamically update virtual locations in real-time with respect to the relative locations of current nearby vehicles. This aims to provide deceptive information about the driving routes to attackers, thereby enhancing location privacy protection.

**FIGURE 10.**
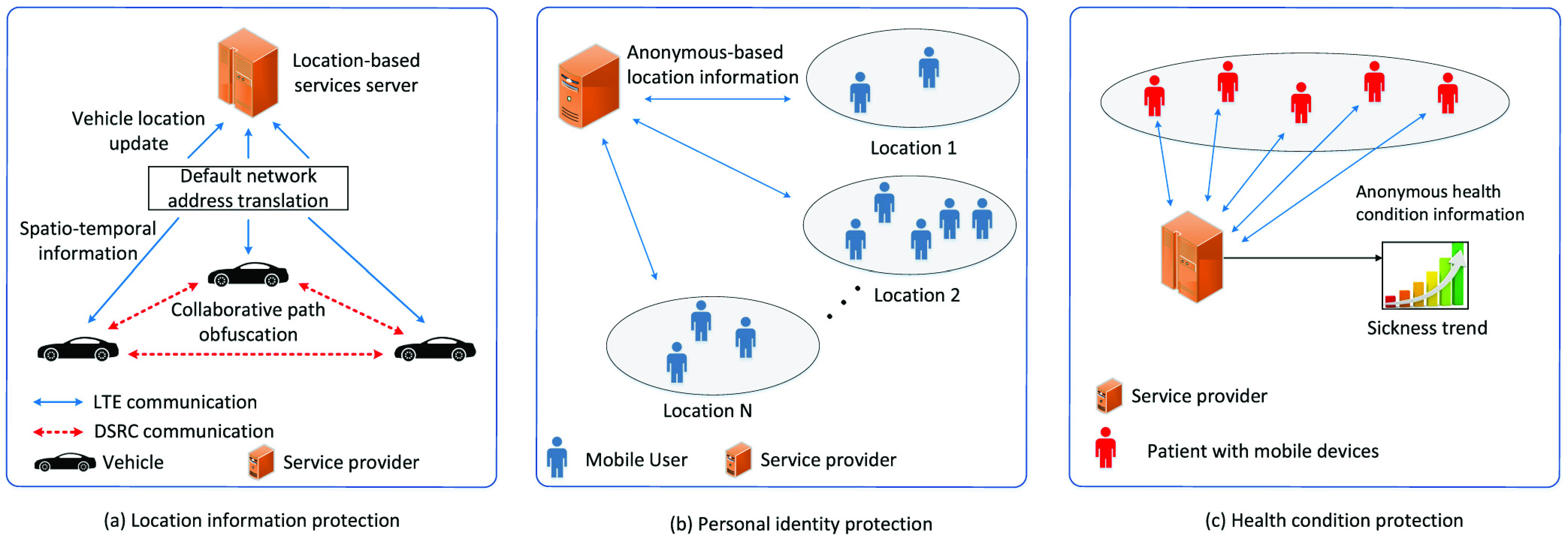
Location-based privacy preserving for social distancing scenarios. In (a) location information protection, the exact location of a vehicle can be obfuscated to protect people’s privacy. To protect (b) personal identity, a user can exchange its identity with nearby trusted users in each location, and thus that user cannot be identified by the attackers. For (c) health-related information protection, the health information can be anonymized.

In addition to the anonymization and obfuscating methods, randomization and perturbation are the methods that can be employed to protect user’s location privacy in social distancing scenarios. In [134], a location privacy-preserving method leveraging spatio-temporal events of mobile users in continuous location-based services, e.g., office visitation, is investigated. Specifically, an $\epsilon $-differential privacy is designed to protect spatio-temporal events against attackers by adding random noise to the event data [137]–[139]. In [140], the authors present a location privacy protection mechanism using data perturbation for smart health systems in hospitals. In particular, instead of reporting the patient’s real locations directly, a processing unit attached to a patient’s body can adaptively produce perturbed locations, i.e., the relative change between different locations of the patient. In this case, the system considers the patient’s travel directions and computes the distance between the patient’s current locations and the patient’s sensitive locations (i.e., patient’s predefined locations which he/she does not want to reveal to anyone, e.g., patient’s treating room). Using this dynamic location perturbation, the need for a trusted third party to store real locations can be removed. Leveraging the aforementioned methods, we can also prevent the service provider from accessing mobile users’ and vehicles’ exact locations/trajectories/paths when they implement social distancing for crowd/traffic density and movement detection. Specifically, a platoon of mobile users/vehicles in a certain area can collaborate together to mix their real locations/trajectories/paths anonymously (Fig. 10(a)). In this way, the service provider will only obtain the aggregated location/trajectory/path information of the platoon instead of each individual’s exact location/trajectory/path for its location privacy.

#### 2) Personal Identity Protection

In addition to protecting mobile users’ location-related information, preserving their personal identities is of importance to improve users’ acceptance of the latest technologies to social distancing. Specifically, we can exchange or anonymize personal identities among trusted mobile users to avoid the attackers identifying the actual identity of each individual user. In [141], the authors develop a pseudo-identity exchanging protocol to swap/exchange identity information among mobile users when they are at the same sensitive locations, e.g., hospital and residential areas. In particular, when a mobile user receives another trusted user’s identity and private key, the mobile user will verify if the encryption of another user’s identity hash function and public key is equal to the encryption of the received private key. If that condition holds, the mobile user will change his/her identity with that user’s identity and vice versa.

Another method to protect personal identity in social distancing scenarios is individual information privacy protection through indirect- or proxy-request as proposed in [142]. In particular, instead of directly submitting a request to the server, a mobile user can have his/her social friends through the available social network resources, i.e., trusted social media, to distribute his/her request anonymously to the server. The request result can be returned to his/her social friends and then forwarded to the requested mobile user, thereby preserving the requested mobile user’s identity. In fact, there may exist some malicious friends who expose the identity of the mobile user. Therefore, the authors in [143] investigate a user-defined privacy-sharing framework on social networks to choose his/her particular friends who are trusted to obtain the mobile user’s identity information. In this case, the mobile user only shares his/her identity information with the particular friends whose pseudonyms match the mobile user’s identity through the authorized access control. Using the same approaches from the above works, we can use local wireless connections, e.g., Bluetooth and Wi-Fi Direct, to anonymously exchange actual location information in a mobile user group, i.e., between a mobile user and his/her trusted nearby mobile users, in an ad hoc way. As shown in Fig. 10(b), when the service provider requires to collect location-related information for the current crowd density detection, a representative mobile user from the group can send the group’s anonymous location information to the service provider, aiming at preserving the personal identity of each mobile user in the group.

Moreover, Apple and Google have recently introduced a key schedule for contact tracing to ensure the privacy of users [3]. Specifically, there are three types of key: (i) tracing key, (ii) daily tracing key, and (iii) rolling proximity identifier. The tracing key is a 32-byte string that is generated by using a cryptographic random number generator when the app is enabled on the device. The tracing key is securely stored on the device. The daily tracing key is generated for every 24-hour window by using the SHA-256 hash function with the tracing key. The rolling proximity identifier is a privacy-preserving identifier which is sent in Bluetooth advertisements. This identifier is generated by using the SHA-256 hash function with the daily tracing key. Each time the Bluetooth MAC address is changed, the app can derive a new identifier. When a positive case is diagnosed, its daily tracing keys are uploaded to a server. This server then distributes them to the clients who use the app. Based on this information, each of the clients will be able to derive the sequence of the rolling proximity identifiers that were broadcasted from the user who tested positive. In this way, the privacy of the users can be protected because, without the daily tracing key, one cannot obtain the user’s rolling proximity identifier. In addition, the server operator also cannot track the user’s location or which users have been in proximity.

Similarly, several solutions have been proposed in [4], [5]. The key idea of these solutions is generating a unique identifier and broadcasting it to nearby devices. In particular, PACT [4] regularly (every few seconds) emits a data string, called chirps, generated by cryptographic techniques based on the current time and the current seed of the user to ensure the privacy. Similarly, in [5], the identifier EphID (called ephemeral ID) is created as follows:

\begin{equation*} \mathrm {EphID} = \mathrm {PRG}\big (\mathrm {PRF}(\mathrm {SK}_{t}, \mathrm {broadcast} {~{}} \mathrm {key})\big),\tag{1}\end{equation*}

where PRF is a pseudo-random function (e.g., SHA-256), broadcast key is a fixed and public string, and PRG is a stream cipher (e.g., AES in counter mode). SK_*t*_ is the secret key of each user during day $t$ which is computed as follows:

\begin{equation*} \mathrm {SK}_{t} = H(\mathrm {SK}_{t-1}),\tag{2}\end{equation*}

where $H$ is a cryptographic hash function. Upon receiving the identifier, other nearby devices will keep it as a log. If a user is diagnosed with the disease, other users who may have encountered the infected person will receive a warning of a potential contact.

With outstanding performance in data integrity, decentralization, and privacy-preserving, blockchain technology can be an effective solution to preserve privacy to enable technology-based social distancing scenarios. A blockchain is a distributed database shared among users in a decentralized network. This decentralized nature of blockchain ensures its immutability property, i.e., the data stored within cannot be altered without the consensus of the majority of network users [147]. Another advantage of blockchain technology is that the users’ anonymity is ensured due to the public-private keys pair mechanism [148]. As a result, blockchain technology can effectively address the personal identity issue in social distancing scenarios where people have to share their movement and location information but not their exact identities. For example, in the infected movement data scenario, we only need to know the movement path of a person, and whether or not that person is infected. In this case, the person anonymity can be ensured with the public-private keys pair mechanism, since there is no way to link the public key to that person’s true identity.

#### 3) Health-Related Information Protection

To monitor the sickness trend in a certain place, e.g., the hospital, for the social distancing purpose (i.e., to inform the upcoming mobile users not to enter a high-risk area/building), the health-related condition information of visiting mobile users has to be shared to provide reliable learning dataset. To protect this highly sensitive information, the authors in [144] propose a differential privacy-based protection approach to preserve the electrocardiogram big data by utilizing body sensor networks. In particular, non-static noises are applied to produce sufficient interference along with the electrocardiogram data, thereby preventing the malicious attackers to point out the real electrocardiogram data.

To provide secure health-related information access for authenticated users, a dynamic privacy-preserving approach leveraging the biometric authentication process is introduced in [145]. Specifically, when a user wants to access the medical server containing his/her health condition, a secure biometric identification at the server for the user’s validity is employed where the exact value of his/her biometric template remains unknown to the server. In this way, the personal identity of the authenticated user can be preserved. To further enhance the anonymity of his/her medical information, the random number that is used to protect the biometric template is updated after every successful login. Then, the authors in [146] propose a secure anonymous authentication model for wireless body area networks (WBANs). Specifically, this framework enables both patients and authorized medical professionals to securely and anonymously examine their legitimacies prior to exchanging biomedical information in the WBAN systems. Motivated by the above works, we can utilize mobile devices, secure service provider, and the aforementioned privacy-preserving approaches to anonymously collect people’s health condition information for illness monitoring in the hospital/medical center (Fig. 10(c)). In this way, the social distancing through monitoring the sickness trend can be implemented efficiently while preserving the sensitive information of the people in the illness areas.

### B. Real-Time Scheduling and Optimization

**FIGURE 11.**
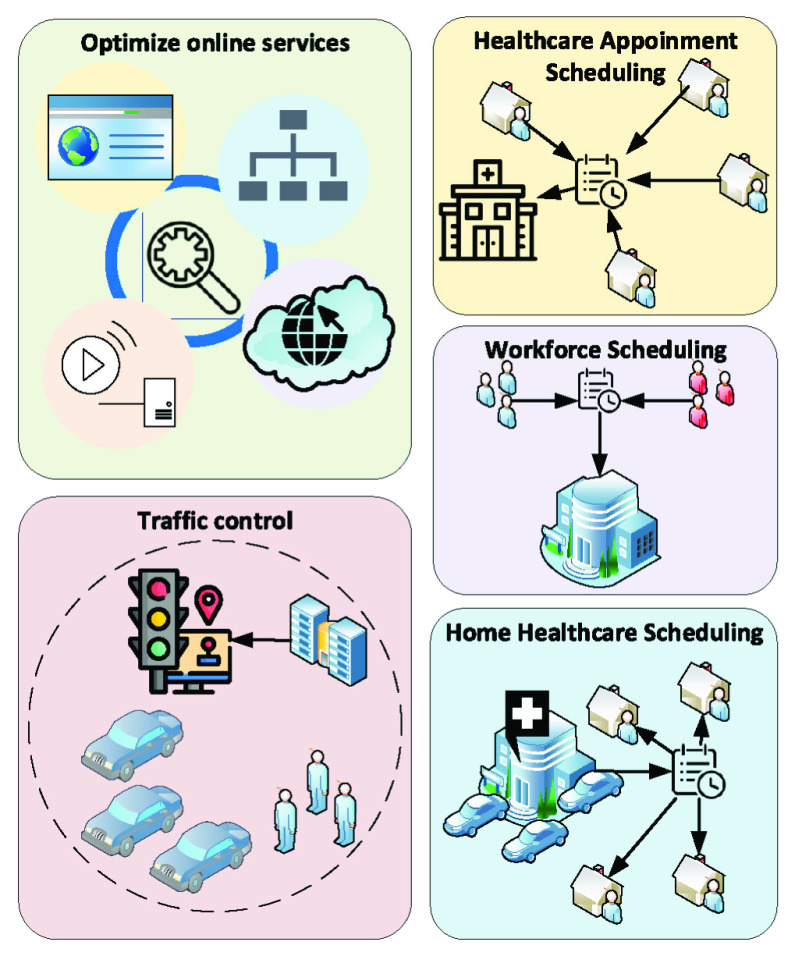
Scheduling and optimization for several social distancing scenarios including reducing the simultaneous presence of employees (workforce scheduling), patients (healthcare appointment and home healthcare scheduling), and traffic (traffic control). Moreover, network resources can be optimized to meet surging demands on online services while more people are working remotely from home.

In the context of social distancing, real-time scheduling and optimization techniques can play a key role in preventing an excessive number of people at a given place (e.g., supermarkets, hospitals) while maintaining a reasonable Quality-of-Service level. Fig. 11 illustrates several social distancing scenarios where scheduling and optimization techniques can be applied. In particular, proper scheduling can help reduce the number of necessary employees at the workplace and the number of patients coming to the hospital, thereby minimizing the physical contacts among people. Moreover, traffic scheduling can help to reduce the peak number of vehicles and pedestrians, and network resource optimization [71] (e.g., network/resource slicing) can meet surging demands on online services while more people are working remotely from home.

#### 1) Workforce Scheduling

Workforce scheduling can help to limit the number of people at the workplaces while ensuring the necessary work is done. While working from home is encouraged in social distancing, some essential work requires people to be present at the workplace for important tasks (e.g., health, transportation and manufacturing). Moreover, different types of tasks impose various constraints such as due date (time constraints), dependence among tasks (precedence constraints), skill requirements (skill constraints), and limited resources usage (resource constraints) which further complicate the scheduling problem. For such scenarios, workforce scheduling techniques can be utilized to optimally align and reduce the number of required employees to practice social distancing. In [72], a novel three-phase algorithm is proposed for workforce scheduling to optimize the operational cost and service level simultaneously. Another Genetic-Algorithm-based hybrid approach is presented in [73], which optimizes the schedules of the workforce according to multiple objectives including urgency, skill considerations, and workload balance. Similarly, in [74], a Mixed-Integer-Programming-based approach is developed to minimize the operational cost with consideration of skill constraints. It is worth noting that the main objective of these approaches is to minimize cost, which is not the highest priority in the context of social distancing. In [75], [76], and [77], several methods are proposed to optimize the workforce schedules with consideration of rotating shifts, which indirectly reduce the number of employees to a certain extent. Nevertheless, the main objective of these approaches is reducing costs. Therefore, developing techniques to reduce the physical contacts or distance among employees at the workplace is critical for workforce scheduling in social distancing scenarios.

#### 2) Medical/Health Appointment Scheduling

Besides workforce planning, scheduling techniques can also help to optimize healthcare services, especially healthcare appointments and home healthcare services, thereby decreasing unnecessary traffic and the number of patients coming to hospitals. Several approaches have been proposed to effectively schedule appointments. In particular, a local search algorithm is proposed in [78] to minimize patient waiting times, doctor idle times, and tardiness (lateness). Moreover, a two-stage bounding approach and a heuristic are presented in [79] and [81], respectively. However, a common limitation of these techniques is that they do not take into account the uncertainties in the duration of the appointments and the possibility that the patient will not come to the scheduled appointment. To address that, the uncertainty in the processing times (e.g., of surgeries) is considered by a conic optimization approach in [80]. Similarly, a multistage stochastic linear program is developed in [82] to minimize patient waiting times and overtime, which takes into account the unpredictable appointment duration and unplanned cancellations. Although there are many effective approaches to optimize appointment scheduling, the open issue is to develop techniques that specifically minimize or control the number of patients simultaneously coming to the hospitals to maintain a suitable level of social distancing, similar to that of the workforce scheduling scenario.

#### 3) Home Healthcare Scheduling

Similar to appointment scheduling, home healthcare services (HHS) can help to reduce the pressure on hospitals and traffic in the social distancing context. In [83], a multi-heuristics approach is proposed for HHS scheduling to minimize the total traveling times of HHS staff. An extended problem is presented in [84], where the objective also includes minimizing the tardiness and additional skills and time constraints are considered. For this problem, local search-based heuristics are proposed in the paper. Another local search-based heuristic is proposed in [85] for HHS scheduling with the objective to minimize traveling times and optimize Quality-of-Service while considering workload and time constraints. In [86], a Genetic-Algorithm-based hybrid approach is proposed for HHS scheduling with uncertainty in patient’s demands to minimize transportation costs. Also addressing uncertainties, a branch-and-price algorithm is proposed in [87] to minimize the traveling costs and delay of services while considering stochastic service times. Unlike in workforce planning and appointment scheduling, HHS scheduling techniques can be more effectively applied to social distancing scenarios because they can minimize the traveling distances while ensuring Quality-of-Service.

#### 4) Traffic Control

Scheduling techniques have also been applied for traffic control. In social distancing scenarios, scheduling techniques can help to regulate the traffic level, especially the number of pedestrians. In [88], a novel scheduling algorithm is developed for traffic control, considering both vehicles and pedestrians, to minimize the delays. Similarly, a macroscopic model and a scheduling algorithm are proposed for traffic control, which jointly minimize both the pedestrians and vehicle delays in [89]. Another scheduling approach is proposed in [90] that considers both pedestrians and vehicles. Different from the previously mentioned approaches, this approach only focuses on minimizing pedestrian delay. Although there is a vast literature on traffic scheduling techniques, the social distancing implications have not been taken into account. For example, to maintain social distancing, a more meaningful objective would be to reduce/constrain the peak number of pedestrians on the street at the same time.

#### 5) Online Services Optimization

When social distancing measures are implemented, more people will be staying at home e.g., working from home. Physical meetings/gatherings will move to virtual platforms, e.g., webinars. That results in much higher Internet traffic and corresponding virtual service demands (e.g., video streaming, broadcasting, and contents delivery). Therefore, optimizing online services delivery is a challenging issue in the social distancing context. Fortunately, online services optimization is a well-studied topic with a substantial body of supporting literature.

For example, in [91], a novel algorithm is proposed to optimize the contents delivery process in a *CDN semi-federation* system. In particular, the algorithm optimally allocates the content provider’s demand to multiple Content Delivery Networks (CDNs) in the federation. The results show that the latency can be reduced by 20% during peak hours. Another technique to reduce the delay and network congestion is edge-caching, which brings the contents closer to the network users (e.g., [92]). In [93], the performance of two edge-caching strategies, i.e., coded and uncoded caching, are analyzed. Moreover, two optimization algorithms are developed to minimize the content delivery times for the two caching strategies.

Besides the contents delivery, the demands on video streaming traffic are also much higher during social distancing implementation because there are many people who work from home. In that context, emerging networking technologies can be an effective solution. For example, an architecture utilizing HTTP adaptive streaming [94] and software-defined networking technology is proposed to enable video streaming over HTTP. Moreover, a novel algorithm is developed to optimally allocate users into groups, thereby reducing communication overhead and leveraging network resources. The results show that the proposed framework can increase video stability, Quality-of-Service, and resource utilization.

Scheduling and optimization are well-studied topics with a vast literature available, which can be utilized for different social distancing scenarios such as workforce, healthcare appointment, home healthcare, and traffic scheduling, and optimization of online services delivery. Nevertheless, except for the home healthcare service scenario, the existing techniques’ objectives do not align with the objectives of social distancing. Moreover, scheduling algorithms are often developed such that they are only efficient for specific problems. Therefore, developing novel optimization/scheduling algorithms in operations research and adopting social distancing as a new performance metric or design parameter is very much desirable. Furthermore, the optimization of Internet-based services such as content delivery can help to encourage people to stay at home during social distancing periods by ensuring the service levels.

### C. Incentive Mechanism to Encourage Social Distancing

Due to the people’s self-interested/selfish nature characteristics in their daily life [163] (especially during the pandemic outbreak), incentive mechanisms can be very helpful in encouraging people to accept or share relevant information to enable new social distancing methods. These mechanisms have been thoroughly discussed in crowdsourcing as implemented in [135], [168]–[171]. Therein, the service providers can provide incentives to a large number of people to attract their contributions in data collection for crowdsourcing processes. For example, the contract theory-based incentive mechanism for crowdsourcing is discussed in [168], [169]. In particular, this approach is considered an efficient mechanism to leverage common agreements between the participating entities, e.g., a service provider and its mobile users, in a certain area under complete and incomplete information from the participants [164]. The use of a game theory-based incentive mechanism to encourage a set of mobile users to form a crowdsourcing community network is investigated in [135], [170]. Then, in [171], the authors utilize an auction theory-based approach incentive mechanism to stimulate mobile users’ participation in crowdsourcing tasks such as traffic monitoring. In the following, we also highlight the existing incentive mechanisms and how they can be further adopted to encourage social distancing applications.

#### 1) Distance Between Any Two People and Distance To/From Crowds

**FIGURE 12.**
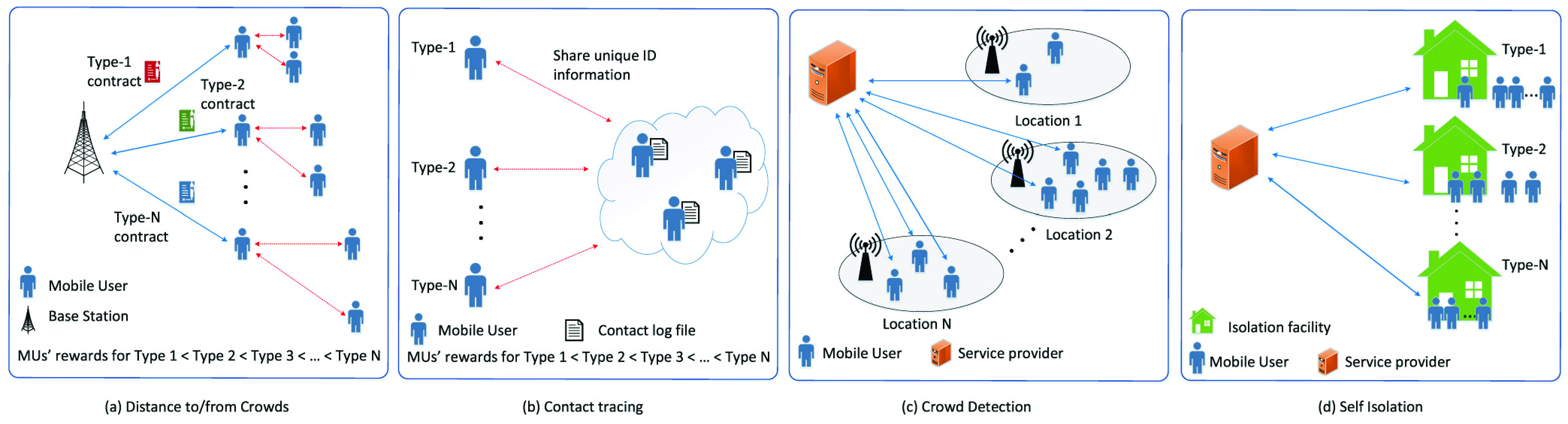
Contract-based incentive design scenarios to encourage social distancing. In (a) distance to/from crowds scenarios, users can be rewarded if they keep a *safe* distance from each other. For (b) contact tracing, users can be rewarded when they share their close-contact information. For (c) crowd detection, users can be incentivized to share current location information to determine the number of people at the same place. For (d) self-isolation incentive, people who spend more time at home can obtain a higher reward.

To motivate people to keep *safe* distances from themselves to others, contract theory-based incentive models via D2D communications, e.g., Bluetooth, Wi-Fi Direct, can be employed. In [165], the authors propose a contract theory-based mechanism to provide a higher reward for D2D-capable mobile users if they send the information to a requesting mobile user with a higher transmission data rate. Taking into account the number of potential nearby mobile users in proximity, the authors in [166] introduce the same mechanism such that a mobile user will receive a higher payment if they can share the information with more nearby users. Likewise, the same approach considering a higher reward for a mobile user who has shorter distances in sharing its information to nearby D2D pairs is presented in [167]. Inspired by the aforementioned works, we can consider the contract theory-based method along with D2D communications to encourage people to keep distances from other people/crowds. Specifically, mobile service providers can be subsidized/funded or requested by the government to provide incentives to their users to keep a distance from others when they are in public. Specifically, a service provider can offer contracts to mobile users, as illustrated in Fig. 12(a). Considering the current distances from the nearby mobile users and capability to inform them through D2D communications, those mobile users can obtain more rewards when they successfully keep a sufficient distance (e.g., at least 1.5 meters) from other people/users. A violation (e.g., getting closer than 1.5 meters to someone) can lead to a “penalty” (e.g., losing part of the previous rewards).

#### 2) Contact Tracing

In a pandemic outbreak, contact tracing is considered one of the most important actions to contain the spread of the disease. To trigger each mobile user for information sharing, e.g., mobile user’s public identity, the network operator requires to offer incentives to those who contribute such information (besides privacy-preserving solutions). In [168], the authors introduce a contract theory-based incentive mechanism in a crowdsourced wireless community network. In particular, the network operator offers contracts to network-sharing mobile users containing a Wi-Fi access price (for their nearby mobile users accessing the network sharing) and a subscription fee (for the network-sharing mobile users). Motivated by this work, we can also develop a contact-tracing framework which allows a mobile user to broadcast his/her public identity to the nearby mobile users as long as their distances are within 1.5 meters. Then, the nearby mobile users can store this public identity in their close-contact log files including the time and location when they receive that public identity as shown in Fig. 12(b). Mobile users who store such log files will pay the sharing mobile user to compensate for the information sharing. In this way, when at least one of the mobile users in the log files is infected by the contagious disease, the mobile service provider can alert the mobile users with the log files to implement social distancing.

#### 3) Crowd Detection

A high density of people in specific areas can make contagious diseases to spread the infection more quickly due to people’s close proximity. To support social distancing, an incentive mechanism approach can also be applied to detect the people density in public areas or the number of people in a building. In [169], the authors present a tournament model-based incentive mechanism to encourage mobile users (with various performance ranks) connected to the local wireless networks, e.g., Wi-Fi hotspots, to send the location and unique identifier of the networks to the service provider (Fig. 12(c)). From the hotspots’ location information, the service provider can then determine the people density in each hotspot area or the number of people in a building (which may have several hotspot areas). Using the above method, we can also encourage mobile users to avoid non-essential public places, e.g., restaurants and shopping malls. In this case, the reward can be adapted according to the locations and essential level of the services (e.g., cinemas, restaurants, grocery stores, schools, and hospitals).

In addition to the people density detection, we can adopt incentive mechanisms to monitor the density of vehicles on the city roads for traffic crowd avoidance purposes. In fact, the contagious diseases, e.g., coronavirus, can remain on the surfaces for four hours up to several days [172]. Thus, avoiding traffic jams on the roads can reduce the possibility of disease infection. In [173], the authors propose a reward-based smartphone collaboration method to support data acquisition for location-based services. Specifically, a client will attract surrounding smartphone users, e.g., vehicular users on a highway, to collaborate together with the aim to build a big database containing location information as implemented in Google’s Android smartphones and Apple’s iPhone [3]. The joining smartphone users then receive shared rewards from the client considering their collaboration costs. Based on this database, the client can determine the traffic levels according to the vehicles’ density on the roads dynamically and sell this information to the authorities or service provider. Such information can be useful for several social distancing scenarios such as crowd detection, traffic/movement monitoring, and traffic control.

#### 4) Location/Movement Sharing and Stay-at-Home Encouragement

To further drive people away from high-density public places, one can also consider incentive mechanisms for better social distancing efficiency (especially for the people with their mobile devices). In particular, the authors in [174] study the uneven distribution of the crowdsourcing participants when maximizing the social welfare of the network. To address this problem, a movement-based incentive mechanism to stimulate the participants to move from popular areas to unpopular ones was introduced. This approach guarantees that the participants will announce their actual costs for further reward processes. Likewise, an incentive mechanism in spatial crowdsourcing considering budget constraints to reduce imbalanced data collection is discussed in [175]. Particularly, the service provider will provide a higher reward when the mobile users are willing to participate in remote locations instead of nearby locations where they belong to (based on their daily routines). A similar work utilizing a redistribution algorithm to incentivize crowdsourced service providers from oversupplied areas to undersupplied ones is also investigated in [136]. The above works are then extended in [176]. Instead of encouraging mobile users to completely move to faraway locations, the service provider will offer a task-bundling containing the nearby and remote tasks for each participating mobile user. All of these works show that the proposed incentive mechanisms can efficiently balance the various location popularity such that we can encourage people to move to low-density places.

In a narrow-down scenario, we can also utilize an incentive mechanism to encourage family-isolation/group-isolation for the possible vulnerable/at-risk people, e.g., sick people and older people. For example, the authors in [177] propose a spatio-temporal-based incentive mechanism using both smartphone and human intelligence in an ad hoc social network. This framework allows a very large crowd to work together in providing information sharing, i.e., geo-tagged multimedia resources, while receiving incentives from the system. Based on this method, we can also engage the vulnerable/at-risk groups to isolate themselves and deliver incentives for them at a certain location during a particular period (Fig. 12(d)). The larger number of vulnerable/at-risk members in a group, the higher incentives will be given. Furthermore, we can design a real-time incentive mechanism to encourage people to implement self-isolation by providing more rewards for those who spend more time at a given location, e.g., at home. In this case, the reward can be negative, i.e., penalty, to discourage people from going to crowded places.

### D. Pandemic Mode for Social Distancing Implementation

An occasional pandemic outbreak in a particular period can drive the mobile service providers, e.g., Google and Apple, to build up a pandemic mode application for current users’ mobile devices, e.g. smartphones. This application represents a comprehensive framework utilizing the current pandemic situation, i.e., infected movement data, to help the mobile users stay aware of the contagious diseases and perform cautious actions to slow down the spread of the diseases through implementing social distancing. To this end, the use of users’ smartphones is very crucial to realize this pandemic mode application as similarly implemented for smartphone-based disaster mode application in [107]–[113]. When a contagious disease outbreak is imminent, the government can first broadcast an urgent notification for mobile users to install/deploy the official pandemic mode application in their smartphones. Then, based on the current infected movement data, e.g., the current reported number of infected people and currently infected areas, from the government officials, the service providers can determine the risk levels of the pandemic and activate a certain level in the smartphones. Considering the risk level, the smartphones can leverage the existing sensors and wireless connections to perform effective contact tracing activity for contagious disease containment.

#### 1) Infected Movement Data

To determine the risk levels of the pandemic mode, the authorities first need to monitor the current infected movement information, i.e., infected areas and the number of infected people. Based on this observation, the authorities then can orchestrate the pandemic mode risk levels and notify mobile users so that they can avoid the areas where the highly-likely infection exists according to the current risk level. In [114], the authors introduce an identification framework to observe the spatial infection spread based on the arrival records of infectious cases in subpopulation areas. Considering susceptible and infectious people movement in metapopulation networks, the framework first splits the whole infection spread into disjoint subpopulation areas. Then, a maximum likelihood estimation is applied to predict the most likely invasion pathways at each subpopulation area. Using a dynamic programming-based algorithm, the framework can finally reconstruct the whole spread by iteratively assembling the invasion pathways for each subpopulation to produce the final invasion pathways. Then, the authors in [115] present a spatial-temporal technique to locate real-time influenza epidemics utilizing heterogeneous data from the Internet. In particular, the technique constructs a multivariate hidden Markov model through aggregating influenza morbidity data, influenza-related data from Google, and international air transportation data. This aims to identify the spatial-temporal relationship of influenza transmission which will be used for surveillance application. Through experimental results, the technique can predict an influenza epidemic ahead of the actual event with high accuracy. Recently, Google and Apple also create a framework to demonstrate the community mobility trend with respect to the COVID-19 outbreak [117], [119]. In particular, this framework is generated based on the regions of mobile users and changes in visits monitoring at various public places, e.g., groceries, pharmacies, parks, transit stations, workplaces, and residential areas.

**FIGURE 13.**
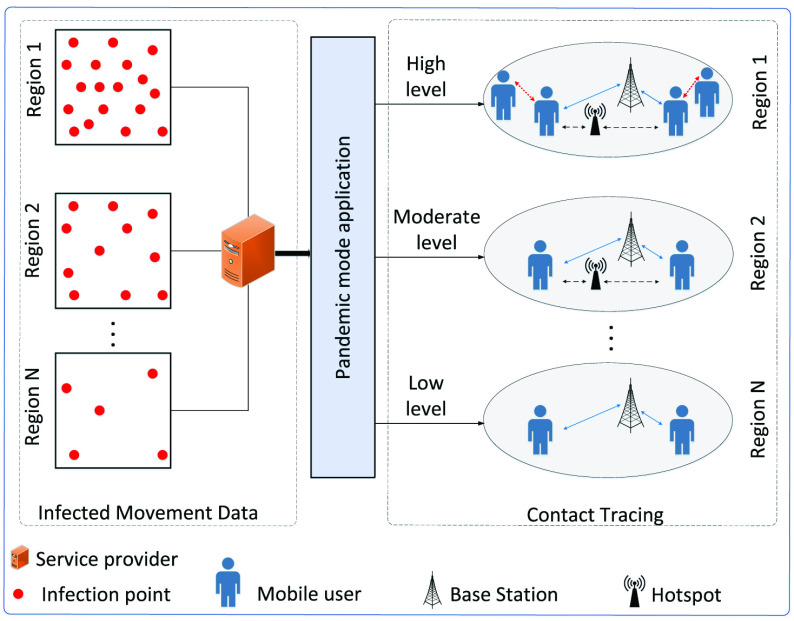
Pandemic mode in future infrastructures to support social distancing. In the infected movement data scenario, the locations of infected cases can be used to determine the pandemic mode risk level of a region. Based on this risk level, the authorities can allow different types of technologies for contact tracing, e.g., low-risk regions only use cellular for contact tracing, whereas high-risk regions can utilize cellular, Wif-Fi, and Bluetooth for contact tracing.

Motivated by the above works, the authorities can first collect the spatio-temporal infectious disease-related information from the Internet and official reports. Using the aforementioned methods, the authorities can then extract meaningful information about the spread locations/pathways and time of the infectious diseases, which leads to various spatio-temporal disease spread levels. Based on these disease spread levels, the authorities can customize the pandemic mode risk level for different regions, e.g., states, cities, and provinces, at different times. For example, if the disease spread level, e.g., the density of infected people, at a particular city is high, the authorities can set the pandemic mode into a high-risk level for a week (as shown in Fig. 13). Otherwise, the pandemic mode level can be set at a low-risk level.

#### 2) Contact Tracing

After determining the risk levels of the pandemic mode based on the infected movement data, the authorities can broadcast the risk level notification through smartphones’ pandemic mode application. Afterward, the smartphones can perform contact tracing to help quickly discovering infected people for efficient outbreak containment [120], [121]. Based on the risk level of the pandemic mode, the smartphones can automatically trace contacts using certain sensors and wireless connections. For example, Google and Apple currently collaborate together to develop a contact tracing application utilizing Bluetooth technology, aiming to quickly detect past contacts among mobile users in close proximity [3]. In this case, the Bluetooth is used to exchange beacon signals containing unique keys between two smartphones prior to storing these keys to the cloud server for infected people notification. Similarly, the work in [122] develops a wireless sensor system to exchange beacon signals between a mobile device with other nearby mobile devices as its contact information. In another work, an epidemiological data collection scheme utilizing users’ smartphones is described in [123]. Specifically, a user’s smartphone can be used as a sensor platform to collect high accurate information including the user’s location, activity level, and contact history between the user and certain locations. Then, a smartphone-based contact detection system leveraging the smartphone’s magnetometer history is investigated in [124]. To determine the close contact, the system measures the linear correlation between two smartphones’ magnetometer records.

Inspired by the aforementioned works, smartphones can be utilized as crucial tools to implement contact tracing considering the current risk level of the pandemic mode activated by the authorities (as illustrated in Fig. 13). In particular, if the authorities activate low-risk levels, i.e., the current number of infected people and areas are small, smartphones can trace close contacts using cellular networks only. In this case, the pandemic mode application will disable certain sensors, Bluetooth, and Wi-Fi by default. However, if the high-risk level pandemic mode, i.e., the current number of infected people and areas are large, is activated, the pandemic mode application will enable all of the wireless connections including Bluetooth, Wi-Fi, and cellular network, as well as relevant sensors automatically to trace contacts faster.

Besides smartphone’s built-in sensors, wearable sensors such as physiological (e.g., respiration rate, body temperature, etc.), audio, video, and inertial sensors, as well as wearable devices (GoPro, smartwatch), can all provide meaningful information [178] for contact tracing. For example, when two persons wearing body sensors networks (BSNs), i.e., sets of wearable sensors attached to the body, making contact with each other, a collaborative BSNs system can be utilized to extract information from the contact. In [179], a framework for computing and data fusion from multiple sensors of different BSNs is proposed. To allow the collaboration between the two BSNs, the authors develop novel mechanisms including inter-BSN data communication, BSN Proximity Detection, BSN mutual service discovery and activation, inter-BSN high-level protocols, and cooperative multi-sensor data fusion. As a result, the framework can detect physical interactions such as handshakes between two persons. Although these wearable systems can provide meaningful and accurate data for contact tracing, they pose a threat to people’s privacy. Therefore, the data from these wearable devices should only be used when a pandemic mode is in effect.

## IV. Conclusion

Social distancing has been considered to be a crucial measure to prevent the spread of contagious diseases such as COVID-19. In this Part II, we have presented a comprehensive survey on how emerging technologies can enable, encourage, and enforce social distancing. For each technology, we have provided an overview, examined the state-of-the-art, and discussed how it can be utilized in different social distancing scenarios. Finally, we have discussed open issues in social distancing implementations and potential solutions to address these issues. We suggested that smart infrastructures (e.g., next-generation wireless systems like 6G, smart home/building, smart city, intelligent transportation systems) should incorporate a pandemic mode in its standard architecture/design. Such an operating mode allows us to better (systematically) respond to COVID-19-like pandemics in the future.
